# Comprehensive Observations of Magnetospheric Particle Acceleration, Sources, and Sinks (COMPASS): A Mission Concept to Explore the Extremes of Jupiter’s Magnetosphere

**DOI:** 10.1007/s11214-025-01249-4

**Published:** 2026-01-27

**Authors:** George Clark, P. Kollmann, J. Kinnison, D. Kelly, A. Haapala, W. Li, A. N. Jaynes, L. Blum, R. Marshall, D. Turner, I. Cohen, A. Ukhorskiy, B. H. Mauk, E. Roussos, Q. Nénon, A. Drozdov, E. Woodfield, W. Dunn, G. Berland, R. Kraft, P. K. G. Williams, H. T. Smith, G. Hospodarsky, X. Wu, J. Hulsman, T. P. O’Brien, M. Looper, K. Sorathia, A. Sciola, A. Sicard, M. Donegan, B. Clare, D. Emmell, J. Wirzburger, D. Sepulveda, L. Roufberg, J. Perry, J. Schellhase, D. Pergosky, E. Able, M. O’Neill, C. Fernandes, D. Chattopadhyay, S. Bibelhauser, S. Kijewski, J. Pulkowski, M. Furrow, C. Feldman, J. Nichols, N. Carr, H. Verma, S. Lindsay, E. Bunce, B. Parry, A. Martindale

**Affiliations:** 1 Johns Hopkins Applied Physics Laboratory, Laurel, MD USA; 2 Center for Space Physics, Boston University, Boston, MA USA; 3 Department of Physics and Astronomy, University of Iowa, Iowa City, IA USA; 4 Laboratory for Atmospheric and Space Physics, Boulder, CO USA; 5 Max Planck Institute for Solar System Research, Goettingen, Germany; 6 Institut de Recherche en Astrophysique et Planétologie, CNRS-UPS-CNES, Toulouse, France; 7 University of California Los Angeles, Los Angeles, CA USA; 8 British Antarctic Survey, Natural Environment Research Council, Cambridge, UK; 9 Mullard Space Science Laboratory, University College London, Dorking, UK; 10 Harvard-Smithsonian Center for Astrophysics, Smithsonian Astrophysical Observatory, Cambridge, MA USA; 11 DPNC, University of Geneva, Geneva, Switzerland; 12 Space Sciences Department, The Aerospace Corporation, El Segundo, CA USA; 13 School of Physics and Astronomy, University of Leicester, Leicester, UK

**Keywords:** Jupiter, Radiation belts, Magnetospheres, Heliophysics mission concept studies, Space missions, Comparative planetary processes

## Abstract

Since the dawn of the space age in 1957, humanity has achieved the remarkable feat of exploring all the planets in our Solar System with robotic spacecraft. This glimpse into the diversity of space environments that make up our Solar System has revealed that no two planetary systems are identical; however, each planet harbors key clues in working toward a more unified and predictive understanding of the basic structure and dynamics of all planetary, and even exosolar, magnetospheres. A common feature found in all strongly magnetized planets are regions of trapped, high-energy charged particles called radiation belts. Dedicated missions studying the radiation belts encompassing Earth have led to major space physics discoveries over the past several decades, but Earth’s magnetosphere exists in a relatively small swath of the parameter space found in our Solar System. To expand that parameter space, we present a mission concept that was reported in the recent National Academies of Sciences, Engineering, and Medicine (NASEM) Decadal Survey to expand the frontiers of Heliophysics in the 2024-2033 decade. The mission concept is called COMPASS, short for Comprehensive Observations of Magnetospheric Particle Acceleration, Sources, and Sinks. COMPASS is a mission dedicated to the exploration of Jupiter’s radiation belts, with an unprecedented suite of instruments covering i) particle species from thermal plasma to 10 tens of MeV electrons and relativistic protons and heavy ions; ii) comprehensive magnetic and electric fields and waves; and iii) dedicated X-ray imaging. COMPASS will enable the scientific community to test existing hypotheses and make new discoveries of how Jupiter’s radiation belts are sourced, accelerated, and lost within such a complex system.

## Introduction

Radiation belts are regions of trapped high energy charged particles and are found at many of the magnetized planets in the Solar System. This fact is quite remarkable, since it implies that particle trapping and acceleration in magnetospheric systems is likely a universal process. Of these known radiation belt systems around the Sun, Jupiter is the most complex and extreme by trapping a large number of particles and accelerating them to ultrarelativistic energies (i.e., >100 GeV ions and > 50 MeV electrons). These characteristics render Jupiter more in line with astrophysical systems, e.g., magnetospheres of pulsars and brown dwarfs (Kao et al. 2023; Climent et al. 2023), where electron synchrotron emissions represent a significant loss process that can be observed remotely from Earth. A recent study by Kao and Shkolnik (2024) suggests ∼15% of brown dwarfs have quiescent radio emissions that appear to originate from radiation belts. Therefore, Jupiter is an ideal and relatively nearby magnetospheric system where we can bridge the knowledge gaps between Earth, planetary magnetospheres, and astrophysical systems.

Despite several missions having been dedicated to studying different aspects of the Jovian planetary system, no observatory has yet been fully dedicated—or sufficiently instrumented—to understanding why exactly Jupiter in many ways acts as the Solar System’s greatest particle accelerator. The Juno mission to Jupiter (Bolton et al. 2017) is our current and best hope at unlocking some of these mysteries, since its evolving polar orbit brings the spacecraft deeper and deeper in the inner (< 6 Jovian radii, or R_J_) magnetosphere. Juno is outfitted with in-situ fields and particle instruments, but they were designed for auroral studies and not able to determine the particle distribution functions > ∼ 1 MeV for electrons and > 10 s of MeV for ions (Mauk et al. 2017). Background measurements from buried detectors within instruments have been modeled with some success (e.g., Becker et al. 2017; Denver et al. 2024) but are unable to provide differential intensities as a function of energy and pitch angle which are basic quantities needed to understand the underlying physics. Missions en route to Jupiter, such as JUICE and Europa Clipper, will not only avoid the core region of the radiation belts but they also lack the instrumentation to resolve the distribution functions of the highest energy charged particles, leaving unresolved fundamental questions about Jupiter’s radiation belts.

Therefore, to make great strides in understanding particle acceleration more generally we must first understand the distinctive and universal processes that drive the most intense radiation belts in the Solar System. This is done by addressing the following objectives: (1) origins: revealing how moon and ring materials contribute to the radiation belts even though they simultaneously limit them; (2) acceleration: discover how Jupiter accelerates charged particles to exceptionally high energies; and (3) loss: reveal the loss processes of relativistic charged particles in Jupiter’s magnetosphere and resulting X-ray emissions. Here, we present the Comprehensive Observations of Magnetospheric Particle Acceleration, Sources, and Sinks (COMPASS) mission concept to explore the “heart” of Jupiter’s radiation belt region. This mission is designed to address the fundamental mysteries in Heliophysics outlined by the broader scientific community (e.g., Roussos et al. 2021, NASEM 2025; Nenon et al. 2021) and will extend what Van Allen Probes has accomplished at Earth to even more extreme environments. We provide a detailed science investigation with objectives and a science traceability matrix in Sect. 2, a high-level and a technical overview in Sects. 3 and 4, respectively, and finally, the mission life-cycle costs, assumptions, and benchmarks in Sect. 5.

## Science Investigation

The fundamental motivation to explore such a hazardous region is to investigate the inner workings of extreme radiation environments with the longer-range goal of bridging knowledge gaps between radiation belt physics at Earth, planetary magnetospheres, and the cosmos (e.g., Roussos et al. 2021; Kollmann et al. 2022; Turner et al. 2023; Nénon et al. 2022). COMPASS will pursue the distinct and universal processes that ultimately sculpt space environments and make great strides in understanding acceleration processes more generally. Additionally, Jupiter’s environment continuously exists in a state that cannot be emulated elsewhere in the Solar System—not even during extreme space weather events at Earth. For example, Jupiter’s magnetic field is ∼20,000 times stronger than Earth’s, which easily traps the observed > 2 GeV ions (e.g., Roussos et al. 2021) and > 30 MeV electrons (e.g., Bolton et al. 2004; de Soria-Santacruz et al. 2016; Kollmann et al. 2018) and is expected to trap and accelerate particles far beyond those energies, i.e., >100 GeV ions and > 50-70 MeV electrons. For reasons not fully understood, charged particles are accelerated and accumulated to those high energies, thus forming Jupiter’s intense radiation belts. The electrons are so energetic and intense that they produce two unique attributes: 1) strong synchrotron radiation that is detectable with radio telescopes (e.g., Tsuchiya et al. 2011; de Pater and Dunn 2003; Bolton et al. 2002; Santos-Costa et al. 2001; Santos-Costa and Bolton 2008), and 2) Jovian electrons that leak out of the system overwhelm Galactic Cosmic Rays (GCRs) observed elsewhere in solar system inside of ∼10 AU (e.g., Baker et al. 1979, 1986; Millan and Baker 2012; Roussos et al. 2021; Nenon et al. 2021).

One theory suggests that gyro-resonant acceleration by whistler waves is likely the prevailing mechanism responsible for Earth’s outer radiation belt (e.g., Horne and Thorne 1998; Summers et al. 1998) and it has been proposed as a viable hypothesis in forming Jupiter’s ultra-relativistic electrons (Horne et al. 2008; Woodfield et al. 2014); however, it remains unknown if this is indeed the prevalent process at Jupiter—and therefore possibly all planetary systems—or if other mechanisms play a more dominant role. For example, recent results from Juno have revealed that electrons over the auroral regions of Jupiter are routinely accelerated to multi-MeV energies and their role in seeding Jupiter’s radiation belts is postulated (e.g., Mauk et al. 2017; Clark et al. 2017; Paranicas et al. 2018).

Another recent study further underscores Jupiter as a unique natural laboratory to explore physics that is otherwise only accessible indirectly. Roussos et al. (2022) observed heavy ion distributions deep in Jupiter’s radiation belts reveal a local source of >50 MeV/nucleon oxygen; a process which appears to have strong parallels to astrophysical acceleration mechanisms (e.g., Doyle et al. 2021). Another study by Li and Fan (2022), suggests that Jupiter’s large mass and cool inner core (compared to the Sun), make it an ideal object in our solar system for capturing dark matter. And if dark matter annihilates it is hypothesized to produce >10 MeV electrons. Search for such signals produced from dark matter in our solar system is a new and exciting topic (e.g., Leane and Linden 2023; Blanco and Leane 2024) that Heliosphysics, Planetary, and Astrophysics communities can tackle together with future missions like COMPASS.

**Fig. 1:**
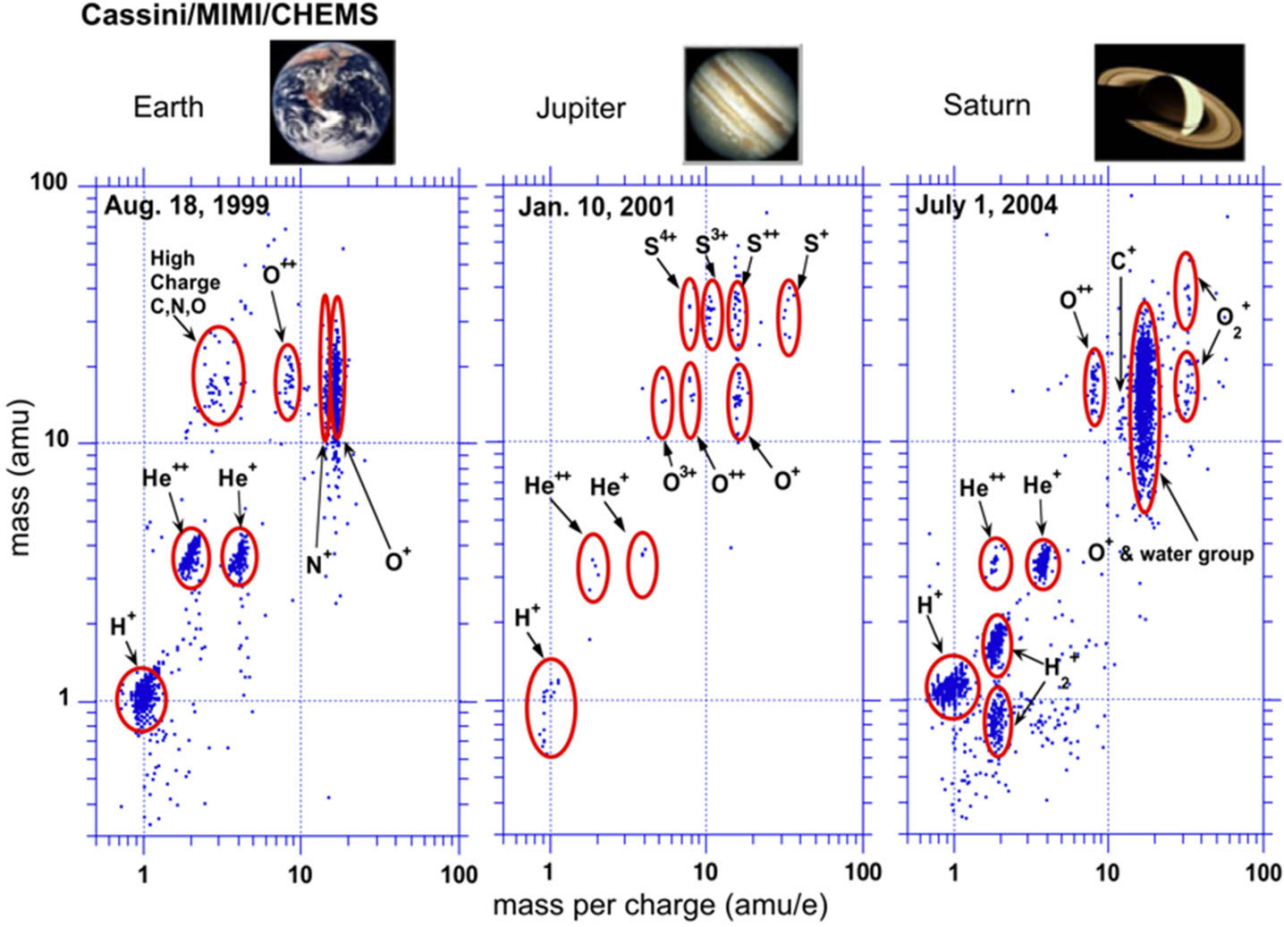
Ion distributions measured by Cassini/MIMI/CHEMS in Earth’s, Jupiter’s, and Saturn’s magnetosphere. The measurements at Jupiter were obtained in its outer magnetosphere during the Cassini flyby. Jupiter’s abundant species goes up to 32 amu. The abundance of ion masses and charge states found at Jupiter make it easier to probe fundamental processes that also exist Earth, i.e., mass vs. charge dependent acceleration (from Hamilton et al. 2005)

Another striking difference between Earth and Jupiter is the source of plasma. Earth’s primary source is external, i.e., from the solar wind, with a lesser contribution from the ionosphere; however, at Jupiter the dominant source is from its geologically-active moon, Io. Io provides roughly 1 ton/s of SO_2_ into the system via interactions between Io’s atmosphere and Jupiter’s plasma environment. SO_2_ dissociates rapidly and becomes ionized via the hot magnetospheric electron population, which results in a multi-species, multi-charge-state plasma (e.g., Allen et al. 2019; Kim et al. 2020; Cohen et al. 2021; Mauk et al. 2004; Hamilton et al. 2005; Clark et al. 2016, 2020a,b). Furthermore, a large species and charge state diversity persists up to ∼100 s of MeV/nuc (e.g., Selesnick and Cohen 2009; Becker et al. 2021; Roussos et al. 2022) The wealth of different particle masses and charge states offers great opportunities to study candidate acceleration processes that respond differently to these quantities (Fig. 1), if future missions are appropriately instrumented to make composition and charge-state measurements (e.g., Artemyev et al. 2020). The global circulation of these energetic ions and electrons through a combination of various candidate transport, acceleration, and loss processes brings them through regions of neutral gas, moons, ring/dust materials and areas of intense plasma waves that scatter particles into the atmosphere. Although many of these mechanisms can act as sinks, the energetic charged particles are able to persevere and form the most intense and energetic radiation belts in the Solar System. Given how strong both particle supply and losses are, it is a mystery why their balances lead to extreme radiation. While several ideas have been developed over the past, all fall far short of appreciating the relative roles of all the competing processes, let alone achieving a predictive understanding.

The examples above form the underlying science theme of the COMPASS mission concept, which is to explore the distinctive and universal acceleration, source, transport, and loss processes that drive the most intense radiation belts in the Solar System. This theme directly addresses 2024-2033 Solar and Space Physics Decadal priorities (NASEM 2025) to explore new environments with the guiding question “what can we learn from comparative studies of planetary systems?”.

### Science Objectives and Traceability

**Fig. 2:**
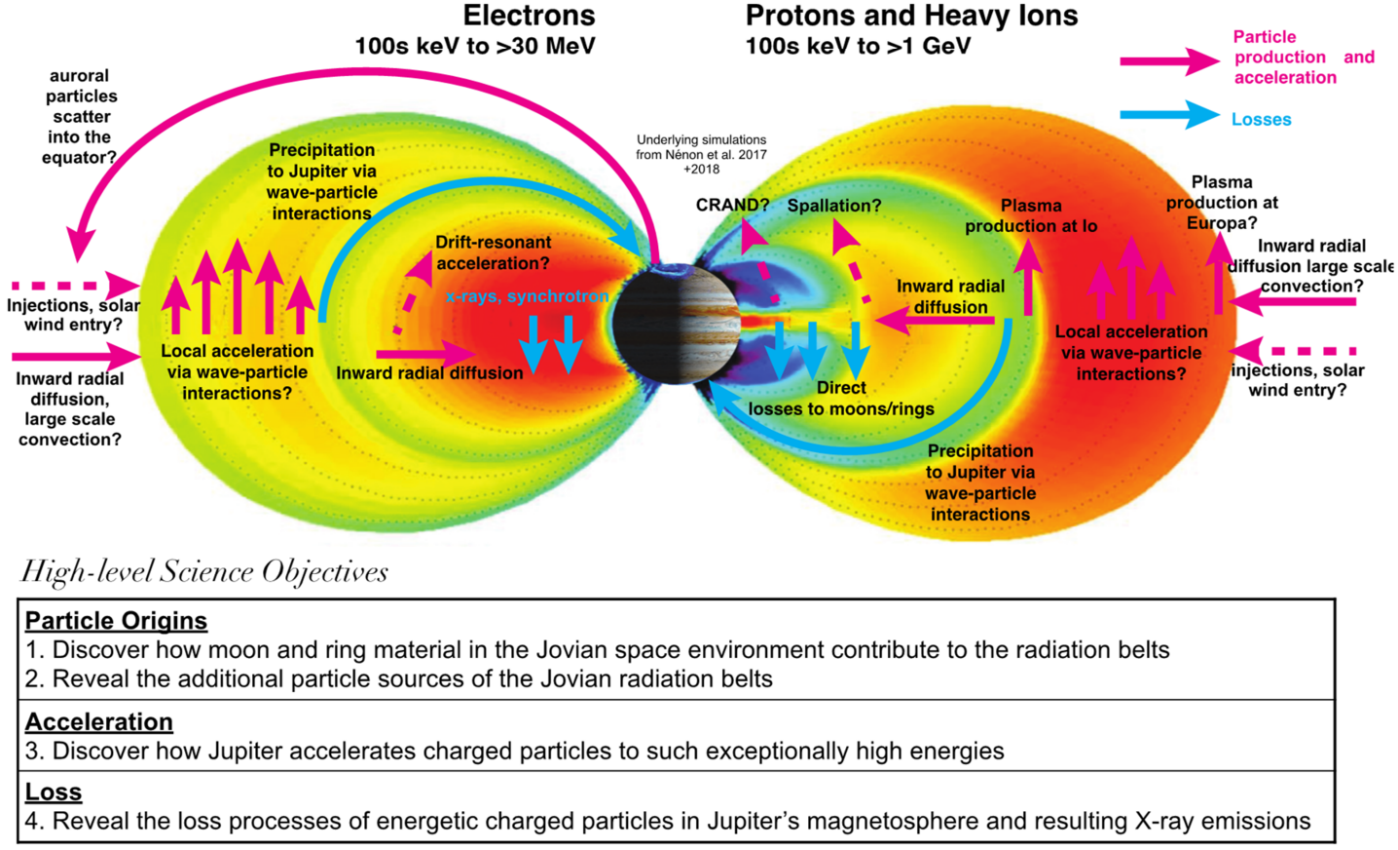
COMPASS will unravel the fundamental mysteries of Jupiter’s radiation belts by addressing several high-level science objectives of significant relevance to the Heliophysics community. Adapted from Nénon et al. (2017) and Nénon et al. (2018)

To make significant progress toward understanding the distinctive and universal processes at play across complex space environments, focused science objectives supported by key questions are critical. This is especially true for Jupiter’s space environment since its large, material-laden magnetosphere with active moons hosts numerous processes that simultaneously facilitate in the production, but also sculpt losses in particle distributions. Therefore, it is necessary to understand how particle origins, acceleration, and loss processes compete across a multi-dimensional parameter space that includes space, time, energy, composition and charge state. The high-level COMPASS science objectives and fundamental mysteries in Jupiter’s magnetosphere are depicted in Fig. 2. These science objectives lead to the following main observational drivers mapped in the Science Traceability Matrix (STM) shown in Table 1: 1) high-fidelity energy- and angular-resolved measurements of the electron and ion populations ranging from thermal energies to > 70 MeV for electrons and to ∼1 GeV for ions; 2) compositional and charge state determination of suprathermal (> 10 keV/Q) ions; 3) AC electric and magnetic plasma wave vectors as well as DC vector magnetic field; 4) novel X-ray imaging of Jupiter’s electron radiation belts and signatures of the interaction of electrons and ions with Jupiter’s atmosphere, plasma and neutral tori, and moon surfaces. Next, we discuss in more detail the science questions necessitated to address COMPASS’ goal.

**Table 1:** COMPASS Science Traceability Matrix (STM). Requirement labels, e.g., MR1 and FR1, are defined in the STM

#### Particle Origins

*Is sourcing from active moons* (*e.g.*, *Io & Europa*) *sufficient to provide seed electron and ion populations to produce and sustain Jupiter’s radiation belts?* Jupiter is known for its magnetosphere filled with ions originating from its geologically active moons, where energetic oxygen and sulfur ion intensities rival those of protons (Mauk et al. 2004; Smyth and Marconi 2006; Smith et al. 2019). Yet, major questions remain even on the origin of the heavy ions. Both Io and Europa exhibit geologic activity (e.g., Roth et al. 2014), but it is unclear which of them is the major oxygen source for the radiation belts. In addition to the moons, the rings in Jupiter’s system might also be a source of heavy ions due to fragments of atomic nuclei being liberated via high-energy particle interactions (Roussos et al. 2021). COMPASS is tailored to measure the species and charge states of ions, which can be compared to physical ion chemistry models to disentangle the roles of Io and Europa from atmospheric processes (e.g., Smith et al. 2019). While moons, their associated neutral gas tori, and rings provide particles to the radiation belts, these objects also simultaneously remove particles through absorption (e.g., Mogro-Campero and Fillius 1976) or cooling from Coulomb collisions (Clark et al. 2014; Nénon et al. 2018). Understanding the balance of sources and losses is absolutely critical in understanding the dynamics of radiation belts (see loss objective).

**Fig. 3:**
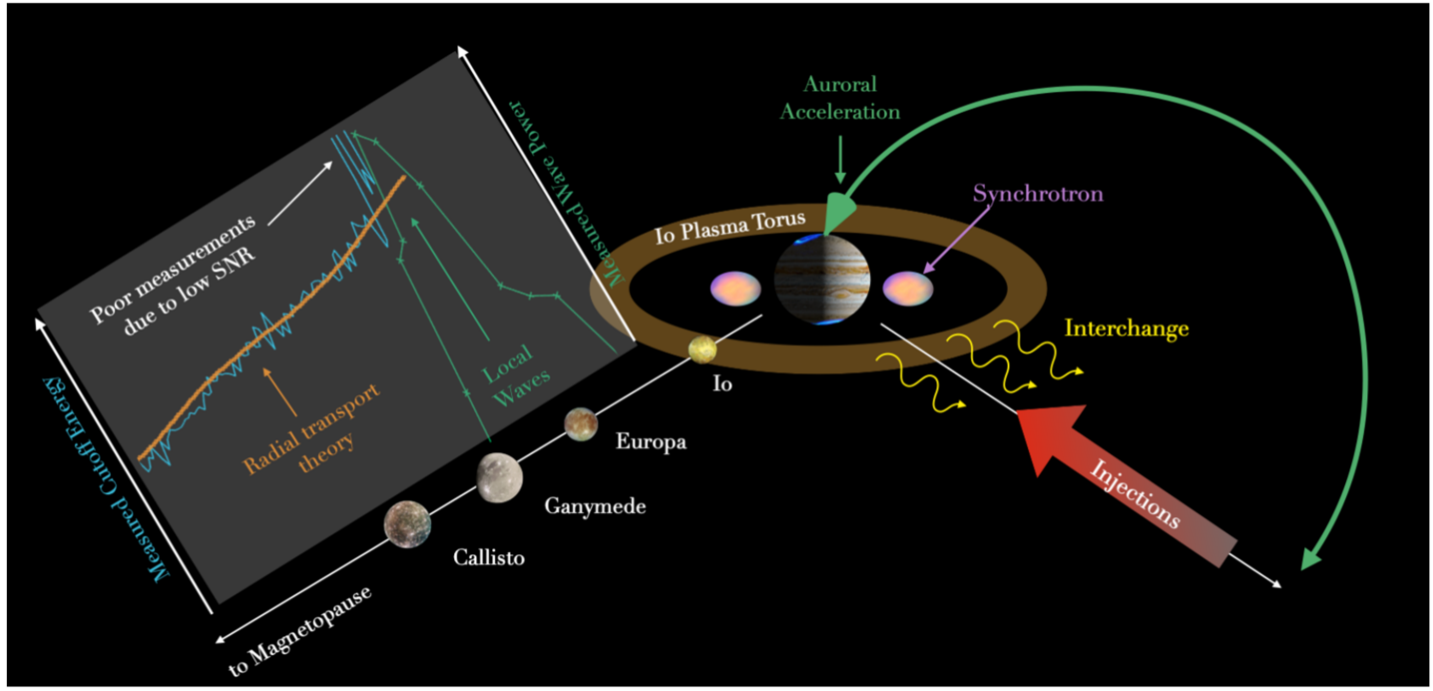
Cartoon depicting several important regions around Jupiter that may contribute to particle acceleration and transport. Not to scale and some regions, i.e., *synchrotron, are purely qualitative and do not depict true spatial extents*

*Can the aurora, solar wind, or atmosphere provide significant particles to the radiation belts?* While a lot of attention in the planetary community was focusing on particle origins related to moons and rings, there is observational evidence that additional processes are at play. For example, the solar wind may gain access to the magnetosphere—a process important at Earth (e.g., Paschmann et al. 1979; Russell 2000; Hasegawa et al. 2004; Wing et al. 2014; Sorathia et al. 2019)— and supply the population of protons and electrons (e.g., Hamilton et al. 1981; Delamere and Bagenal 2010). Moreover, auroral regions of both Earth and Jupiter are known to be sources of energetic ions and electrons. Possibly unique to Jupiter, auroral populations are routinely accelerated to energies > several MeV (e.g., Mauk et al. 2017; Paranicas et al. 2018; Clark et al. 2017). Jupiter’s magnetosphere is also filled with MeV electrons out to the magnetopause region (e.g., Van Allen et al. 1974; Kollmann et al. 2018) suggesting that the original field-aligned particles accelerated in the auroral region may be scattered and end up supplying the equatorial radiation belts (e.g., Speiser 1965; Young et al. 2008; Roussos and Kollmann 2021; Fig. 3). Finally, Jupiter’s atmosphere can also produce charged particles via the Cosmic Ray Albedo Neutron Decay (CRAND) process, where protons and electrons are produced from interactions between Galactic Cosmic Rays (GCRs) and Jupiter’s mostly hydrogen atmosphere (Blake and Schulz 1980; Nénon et al. 2018). This process is observed at Earth (e.g., Selesnick et al. 2014; Li et al. 2017) and Saturn (e.g., Cooper and Simpson 1980; Blake et al. 1983; Cooper 1983; Kollmann et al. 2013, 2022), but its significance at Jupiter has not been proven. COMPASS can distinguish these processes by observing the angular distribution of ions and electrons mapping to Jupiter’s auroral zone. Additionally, COMPASS is tailored to measure the species and charge states of ions which can be compared to physical ion chemistry models to disentangle the roles of Io and Europa from atmospheric processes (e.g., Smith et al. 2019).

#### Particle Acceleration

**Fig. 4:**
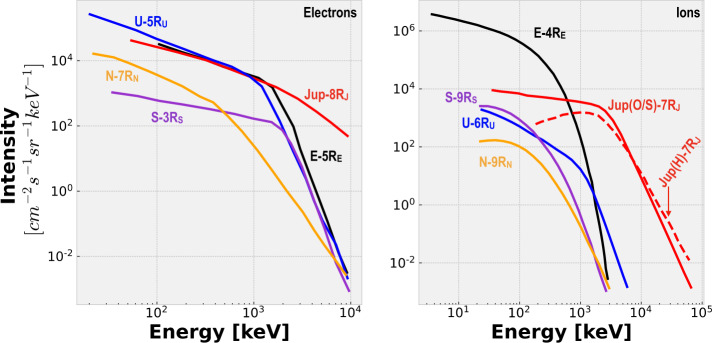
Comparative energetic electron (left) and ion (right) spectra for Earth (E), Jupiter (J), Uranus (U), Saturn (S), and Neptune (N). Ion spectra are for ionized hydrogen (H^+^), unless otherwise noted, e.g., Jupiter spectra for oxygen and sulfur ions (O/S) are also shown. Radial positions for each spectrum, in units of planet’s radius, are shown. Electron spectra are from Mauk and Fox (2010) and ion spectra are from Mauk (2014)

*What processes are responsible for accelerating ions and electrons to such exceptionally high energies in Jupiter’s radiation belts and magnetosphere?* The acceleration processes at Earth are also operating at Jupiter, i.e., radial transport & wave-particle interactions, but their relative significance may be very different. Figure 4 illustrates comparative electron and ion spectra for Earth, Jupiter, Uranus, Neptune, and Saturn and highlights Jupiter’s ability to accelerate particles to much higher energies. The fact that particle production and acceleration can overcome Jupiter’s material-laden magnetosphere that absorbs and cools charged particles, and still greatly exceed the energies and intensities found in any other planetary environment is one of the biggest mysteries in Heliophysics (e.g., Mauk et al. 2004). What makes Jupiter so compelling as a natural laboratory is that it is likely easier to disentangle the interplay between the different acceleration processes found at Earth, even though Earth’s space environment is easier to access and less risky in terms of radiation effects. That is because at Earth, local acceleration occurs over a broad range of radiation belt L-shells (e.g., Li and Hudson 2019; Horne et al. 2005; Thorne et al. 2013; Baker et al. 2014; Shprits et al. 2008; Reeves et al. 2013; Ma et al. 2018; Boyd et al. 2018); however, strong wave activity in Jupiter’s magnetosphere is found near the Galilean moons (Fig. 3) (e.g., Menietti et al. 2021). Note that our picture of plasma waves elsewhere is incomplete due to limited coverage, especially inside of Io’s orbit. Acceleration from radial transport may prove to be a dominant process, which arises from inward radial diffusion (e.g., Kollmann et al. 2018) driven by: random field fluctuations in the magnetosphere (e.g., Saur 2021) or the ionosphere (e.g., Lejosne and Kollmann 2020), centrifugally driven interchange (e.g., Mauk et al. 2002), or large-scale coherent transport (e.g., Hao et al. 2020). Non-adiabatic transport may occur during reconnection in the Jovian magnetodisk and/or magnetotail (e.g., Vasyliunas 1983; Vogt et al. 2010, 2020) or at low altitudes (Masters et al. 2021), leading to acceleration processes that are in principle similar to those found in Earth’s magnetotail (e.g., Turner et al. 2021; Cohen et al. 2021). One of the major thrusts of COMPASS is to *cleanly*—meaning high signal to noise through whatever means necessary—measure energy- and pitch-angle-resolved differential 1 MeV to > 50 MeV electron fluxes, 1 MeV to 1 GeV proton fluxes, and 1 MeV/nuc to > 1 GeV/nuc heavier ion fluxes in conjunction with a full spectrum of plasma wave measurements. This is absolutely essential to the success of understanding Jupiter’s mysterious radiation belts.

#### Particle Loss

**Fig. 5:**
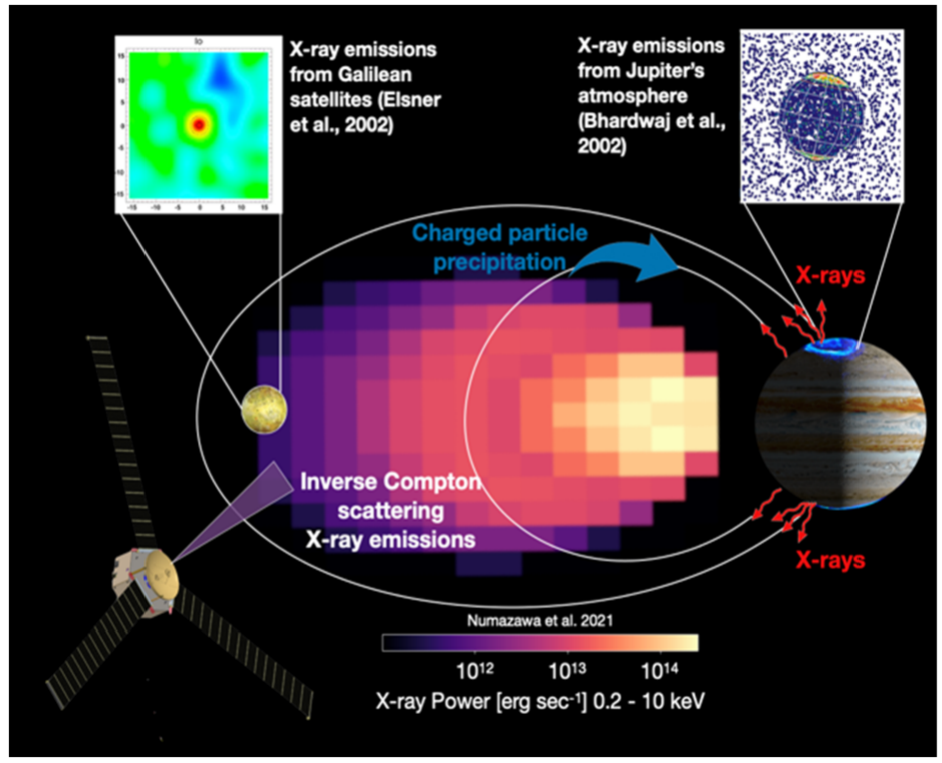
Loss processes of energetic charged particles and inverse Compton scattering regions within Jupiter’s magnetosphere that contribute to X-ray emissions

*Do precipitation losses to the Jovian atmosphere and collisional losses to moons and ring materials balance and ultimately limit Jovian radiation belt intensities?* While acceleration and source processes get a lot of attention in radiation belt physics, losses are similarly important because without them, intensities would accumulate indefinitely. As at Earth (e.g., Marshall and Cully 2020), Jupiter loses particles via precipitation to the atmosphere, but unlike Earth where losses to the magnetopause are important, Jupiter’s standoff distance is located too far away (60-100 R_J_) for this to play an important role. Therefore, losses in the inner magnetosphere are likely the critical factors in sculpting the particle distributions. The radiation belt regions along with the 3 innermost Galilean moons, neutral & plasma tori, and rings are all embedded deep within Jupiter’s inner magnetosphere (L ≤ 15 R_J_). Sparse observations and simulations have shown that wave-particle interactions near Io (e.g., Nénon et al. 2017, 2018; Sulaiman et al. 2020; Szalay et al. 2018) can locally pitch angle scatter ions into the atmospheric loss cone. Moons can also directly absorb charged particles, which in turn also weather the moons’ surfaces, but the efficiency of the process is dependent on where the moon is located at any given time as well as the pitch angle distributions of electron and ions (e.g., Paranicas et al. 2012; Nordheim et al. 2018). Therefore, material-laden magnetospheres such as Jupiter’s provide us with a natural laboratory to probe competing processes acting both as sources and sinks. These loss mechanisms (Fig. 5) are expected to have corresponding signatures in both charged particle distribution functions and in the intensity and spectra of remotely sensed X-rays (e.g., Millan et al. 2002; Marshall et al. 2020; Ezoe et al. 2010; Numazawa et al. 2021)**,** but limitations in the existing in-situ energetic particle measurements from Jupiter’s inner radiation belts and the lack of close-proximity X-ray observations of the Jovian system, prevent us from reaching concrete interpretations about the significance of different radiation belt loss processes. Additionally, electron and ion losses to Jupiter’s atmosphere and moons produce hard (> ∼2 keV) and soft (< ∼2 keV) X-rays (e.g., Gladstone et al. 2002; Branduardi-Raymont et al. 2010; Bhardwaj et al. 2007; Elsner et al. 2002; Dunn et al. 2017; Nulsen et al. 2020). A major design consideration for COMPASS is to enable an unprecedented view of Jupiter’s magnetosphere via X-rays. An X-ray imager configured on a Jupiter orbiting spacecraft can achieve ∼10^7^ more photons over Earth-orbiting assets with unprecedented angular resolution. Jupiter’s intense radiation belts necessitate a mission design with long orbital periods; however, remote observing campaigns with X-rays can monitor the dynamics of the magnetosphere via interactions with moons, neutral tori, photons (via inverse Compton scattering), atmosphere, and rings and thus probe timescales unattainable otherwise. X-ray observations in Jupiter’s material-laden environment will reveal the dynamics of high-energy electrons and ions much like energetic neutral atoms have been used to probe the global dynamics of Earth’s magnetosphere via ion-only interactions with neutral materials. COMPASS’s first science phase (more details on that in the following sections) is also tailored to enable near-simultaneous X-ray observations of Jupiter’s atmosphere connected to COMPASS’s magnetic footprint to probe not only correlations, but also causality.

#### Enabling Unknown Discoveries

**Fig. 6:**
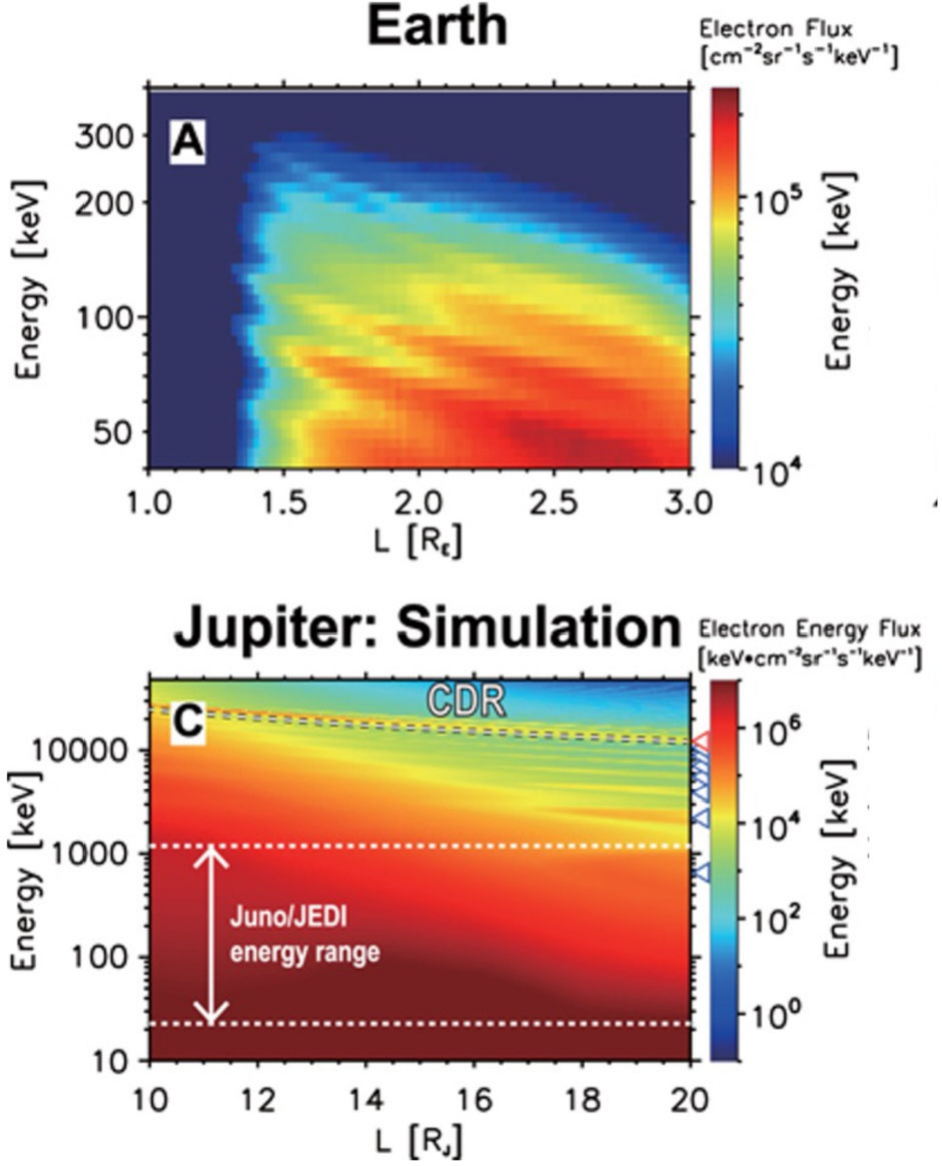
Corotation drift resonance (CDR) driven energy banding as measured at Earth (top panel) and simulated at Jupiter (bottom panel). Results are from Hao, Sun, Roussos et al. (2021). COMPASS can enable meso-to-micro scale measurements using a novel burst data acquisition and downlink plan

Missions to deep space are typically severely downlink limited and therefore heroic efforts are required to reduce data volume while also ensuring mission success. As a result, high resolution data products are either not employed or severely limited in scope (i.e., region or duration), but we know, all too well, the success stories and discoveries enabled from Earth missions downlinking high-resolution “burst” data. NASA’s Magnetospheric Multiscale (MMS) mission is a prime example of a mission making revolutionary discoveries associated with magnetic reconnection in part because of its combined burst data acquisition and scientist in the loop (SITL) function, where selections are made by experts on the ground based on various parameters of interest. To enable the same discovery-level science that is unprecedented in deep space missions, COMPASS made design considerations (i.e., power, communication, and dedicated downlink phases; see Sect. 4) that will enable continuous downlink of burst data inside of Ganymede’s orbit and selective regions outside. Simulations of Jupiter’s magnetosphere (Hill 2017, see Fig. 6) suggest fine structure within the radiation belts is likely and may be analogous to the so-called Zebra stripes discovered in Earth’s radiation belt (Ukhorskiy et al. 2014). Therefore, by enabling very high-energy data collection and downlink, COMPASS has the ability to reveal unknown mesoscale to microscopic processes operating in Jupiter’s radiation belts and broader magnetosphere. Mission success does not need to depend on downlinking burst data and as a result this is one descope option that provides cost (i.e., smaller high-gain antenna, lower utilization of power) and complexity (i.e., SITL and operations) savings.

## High-Level Mission Concept

### Overview

The Johns Hopkins University Applied Physics Laboratory’s (JHUAPL) led the engineering development of the COMPASS mission concept that implements the science objectives discussed in Sect. 2. APL’s concurrent engineering (ACE) laboratory fosters real-time interaction between scientists, instrument developers, and flight system engineers. This interaction allows the team to: i) focus quickly on trades and critical factors in the design to arrive at a concept representing a mission point design at Concept Maturity Level (CML) 4, ii) understand trades and development to be conducted in subsequent mission phases, and iii) identification of mission-level risks and mitigations. The result of this process is a well-defined, feasible mission that accomplishes science goals at reasonable cost and with low schedule risk. The mission concept presented here is the result of trade studies that optimized the mission with regard to factors such as science objectives, concept study requirements, Jupiter’s space environment and engineering constraints, and risk. The end result is a CML 4 point solution that demonstrates COMPASS is a feasible Solar Terrestrial Probe (STP) mission for exploring Jupiter’s extreme magnetosphere.

The main mission and spacecraft design features can be summarized as:

1. A single spacecraft mission with potential launch dates occurring every year, on a $\Delta $V-EGA trajectory to Jupiter with launch energy C3 ≤ 52 km^2^/s^2^
2. Earth-pointed, spin-stabilized spacecraft with 1456 kg dry mass, 3086 wet mass at launch, including a 123 kg science payload
3. Powered through 72 m^2^ Roll-Out Solar Arrays (ROSAs) arranged in three wings to provide 500 W (EOL including margin) at Jupiter
4. Blowdown monopropellant chemical propulsion system to provide 1500 m/s for a Deep Space Maneuver (DSM) that enables transfer to Jupiter, Jupiter Orbit Insertion (JOI) maneuver, Perijove Raise Maneuver (PRM), and science tour $\Delta $V, as well as propellant for statistical trajectory correction, attitude control of the spacecraft, and deorbit maneuver
5. X-band uplink and downlink to provide 230 Gbits of total mission science data return
6. Mission Operations Center/Science Operation Center ground systems to perform all functions needed to operate the mission, return data through the Deep Space Network, distribute science and engineering data to the science teams, facilitate SITL, and analyze and archive mission data
7. The major mission phases are: 1) launch and interplanetary cruise, 2) capture into the Jovian system, and 3) multi-phased science tour that includes disposal via Jupiter impact. More details are found in Sects. 3 and 4.
8. Science phases to mitigate radiation risks and maximize science return:
  8.1 Science Phase I: A high-inclination phase with perijove (PJ) near Io’s orbital distance (5.9 R_J_) critical for addressing the science objectives pertaining to particle origins and losses and optimal for novel remote sensing payloads
  8.1 Science Phase II: A low-inclination phase with PJ ∼ 1.5 R_J_. The primary objective in this phase is to make several deep dives into the heart of the radiation belt and synchrotron region near the magnetic equator. This phase is optimal for in situ payloads and akin to NASA’s Parker Solar Probe mission, where several deep dives are used to unlock the Sun’s mysteries.
9. Total Integrated Dose (TID) < 100 krads behind 2.6 cm Al over the full course of the mission
10. Cost: $FY22 1.2B including Phases A-F, 50% reserves, and Falcon Heavy Expendable launch vehicle

### Navigating Jupiter’s Intense Space Environment

COMPASS is intended to explore the extremes of Jupiter’s magnetosphere. Along with the typical spacecraft thermal environment, any mission to Jupiter must consider the effects of trapped energetic charged particle radiation on spacecraft systems. This is particularly important for COMPASS as the spacecraft will be making in situ measurements of this environment in regions where the charged particle environment is most severe. Therefore, we prioritized understanding and mitigating radiation effects on the spacecraft and payloads as a major design factor in developing this concept. This consisted of several steps: i) early linkage of mission design and the charged particle environment. Simplified models of radiation effects on the spacecraft were used as inputs into trajectory trades to optimize the mission return and impacts on spacecraft design; ii) use of surrogate spacecraft design in radiation analyses to optimize shielding mass estimates; iii) validation of standard charged particle models through comparison with more recent models to understand uncertainties and optimize margins to account for these uncertainties; iv) consideration of shielding trades in the spacecraft design to optimize constraints such as: required shielding mass, mitigations for charging effects, spot shielding, shield vaults, etc. and v) consideration for instrument placement to minimize radiation effects on payloads and data quality.

**Fig. 7:**
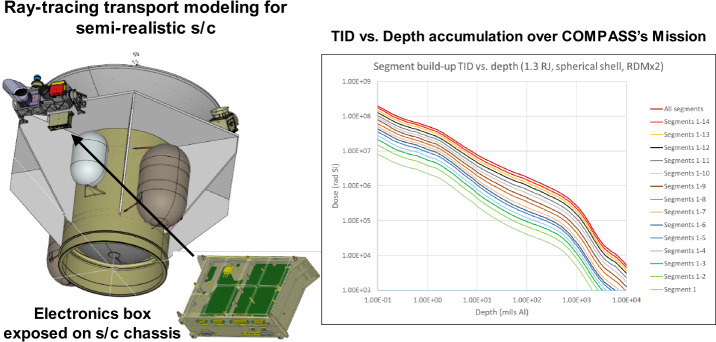
Ray-tracing transport modeling with semi-realistic surrogate s/c & electronics box (left panel). TID vs. Depth accumulation over the prime COMPASS mission for different shielding thicknesses. Dose falls quickly at large thicknesses. “Segments” refer to ranges of orbit numbers. Total TID behind 100 mils Al over all orbit segments is ∼1.8 Mrad, accounting for the standard radiation design margin (RDM) of a factor of 2. The actual shielding will be ∼10 times thicker and reduce dose to between 100 to 300 krad for electronic components at EOL**.** This approach is similar to NASA’s Europa Clipper mission

The result of this analysis is a design that assumes a 100 krad Total Ionizing Dose (TID) requirement for electronic components—with shielding used to reduce levels inside electronic enclosures. Shielding, defined here, can take the form of a vault(s) that contains nearly all electronics, with spot shielding implemented where necessary, i.e., for electronics that must reside outside the vault. In this concept, we provide conservative mass estimates for shielding that we expect will encompass not only the current design, but future designs as the concept matures. Note that many components are available that can withstand higher TIDs, e.g., ratings up to 300 krad, therefore it is reasonable to rely on spot shielding lower TID components to reduce the overall shielding mass. Figure 7 (left panel) illustrates a surrogate spacecraft and data processing unit (DPU) used in a 3-dimensional radiation model. We designed the COMPASS shielding to the GIRE/Grid3 model – an industry standard for radiation analysis (e.g., de Soria-Santacruz et al. 2016) – under the standard assumption of shielding through spherical shells. Figure 7 (right panel) shows how dose can be reduced through increased shielding. It can be seen that 1700 mil of Al are needed to keep 100 krad parts within specification and 1000 mil for 300 krad. Figure 7 also illustrates how the dose accumulates over the various orbits. Our assumptions are very conservative because there are various reasons why the actual dose can be expected to be lower. The state-of-the-art physic-based model JOSE/Salammbô model (Nénon et al. 2017, 2018) is predicting doses that are 60% and 50% lower at 100 and 600 mil of shielding, respectively. Also, the assumption of a spherical shell neglects shielding from the spacecraft body. When assuming a relatively exposed box with 100 mil Al shielding on an approximation of the COMPASS spacecraft (we used NASA’s Io Volcano Observer (IVO) mission concept in this case) on a COMPASS orbit, we find reductions of 30-50%, depending on the location within the box.

Further reductions are possible through selection of the shielding material. While Al yields the highest reduction behind 100 mil, tungsten can reduce dose by additional ∼50% at a thickness equivalent to 1000 mil Al. In combination, all these effects might reduce the dose by an order of magnitude, which provides ample margin.

There are a number of additional considerations for the Jovian environment. For example, the proton component of Jupiter’s radiation belts is expected to require a thick cover-glass (i.e., 500 $\mu $m of borosilicate-type glass called CMG) for the solar arrays in order to prevent unacceptable degradation to their performance. For Spectrolab XTJ Prime solar cells (that approximate the planned Redwire ROSAs) with 500 $\mu $m CMG we expect a charged particle fluence equivalent to of 1.17×10^15^ (1 MeV electrons)/cm^2^. This fluence will lead to roughly a 25% degradation for solar cell maximum power at end of life. Our solar cells were scaled accordingly. Therefore, all of these effects, while challenging, can be successfully mitigated with a rigorous systems engineering approach that includes: trajectory design, shielding mass allocation, electronic parts selection, design decisions, and test and analysis for verification.

### Planetary Protection

Europa is of significant interest because of the processes that may lead to forms of chemical evolution or the origin of life, and any contamination could severely compromise future investigations. For that reason, flyby and orbiter missions to the Jovian system much take the necessary precautions to avoid collision. We show in Sect. 4.11, that the COMPASS tour design carefully considers planetary protection guidelines and disposes the spacecraft into Jupiter. Therefore, COMPASS poses little-to-no risk to Europa concerning planetary protection due to careful mission design ensuring no intersection between the spacecraft and Europa orbits prior to Jovian atmospheric entry at end of mission (see further details in Sect. 4.11).

### Technology Maturity

We assessed Technology Readiness Levels (TRLs) for spacecraft subsystem elements and instruments in the development of the COMPASS concept and it was determined that this mission can be executed with very little technology development since all components of the spacecraft included in the design are at TRL 6 or higher. The instruments included in the concept payload all are based on previously flown instruments that may not represent the state-of-the-art at the time of mission development, but would allow the mission to be flown now without technology development. That being said, technology development areas that would enhance the science return of COMPASS in all areas are recommended. Instrument and subsystem TRL assessments are included in the detailed flight systems.

### Key Trades

We assessed options for all major design decisions and selected the best approach for the mission concept using a combination of mission performance requirements and engineering judgement of the technical benefit, cost, schedule, and risk trade-offs. Major system and subsystem design decisions are described in Table 2.

**Table 2:** COMPASS key mission design trade matrix

Area	Trade Study	Results/Rationale
Data Return	Antenna size, RF power, frequency band, data collection plan	• High data collection rate in Phase 2 of science mission drives required static, non-deployable HGA size and RF power. • Ka-band system requires tight pointing requirements that may not be achievable. X-band chosen to reduce propellant needed and burden on attitude control.
Attitude Control	3-axis vs spin stabilized control. Thruster control vs reaction wheels	• Spin stabilized control chosen to reduce system complexity. 3-axis mode not needed to complete science objectives. Spinning is required to complete science objectives. • Reaction wheels not needed for control as spin-stabilized system is passively controlled.
Solar Arrays	Rigid solar arrays vs Roll-Out Solar Arrays (ROSAs)	• ROSAs selected due to packaging constraints in launch vehicle fairing. • Three panel design chosen for ease in balancing the spinning spacecraft.
Trajectory	Multiple options for trajectory in primary science phases	• Trajectory chosen to minimize radiation exposure and meet science objectives and required measurement locations. • Highest radiation exposure moved to last orbits to maximize probably of success.
Launch Vehicle	Multiple LVs considered	• Only SpaceX Falcon Heavy Expendable launch vehicle (LV) meets requirements for spacecraft mass. • 5 m fairing chosen to accommodate spacecraft design constraints.

## Technical Overview

### Payload Description

**Fig. 8:**
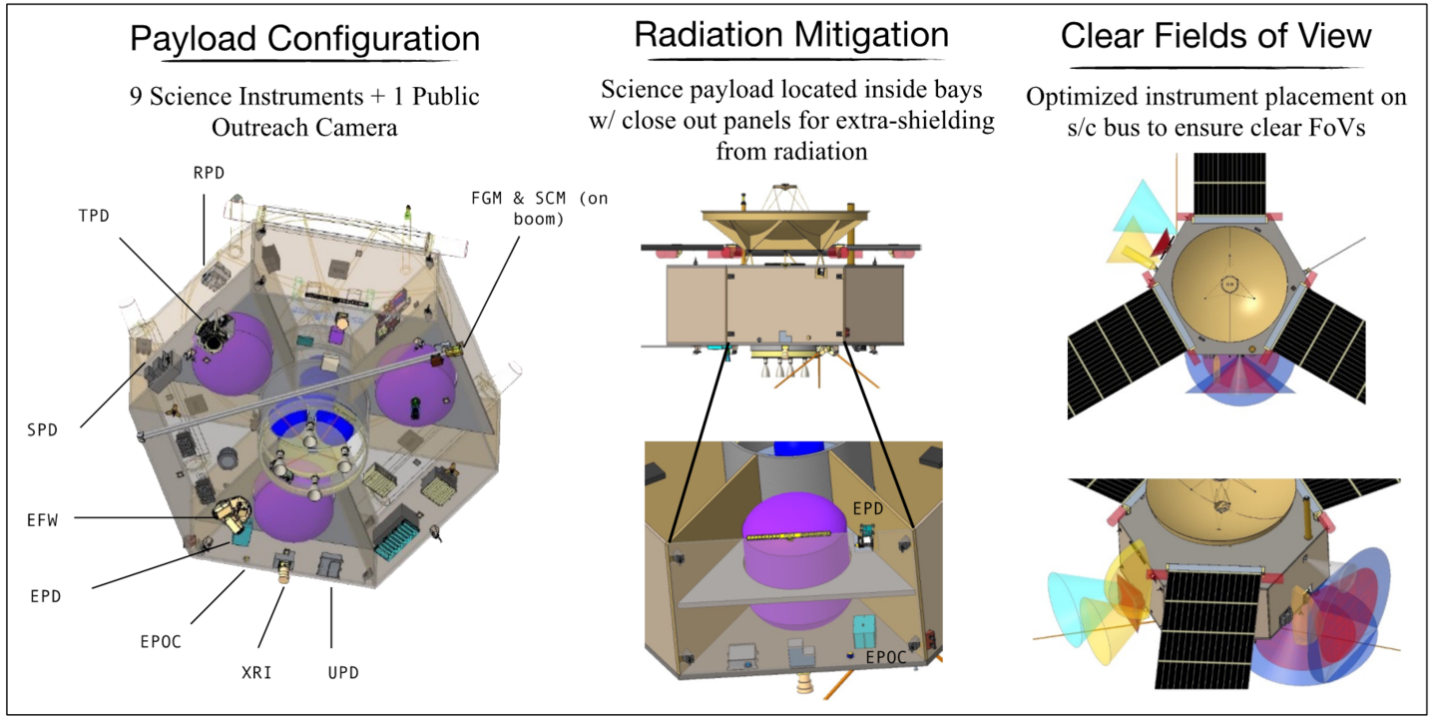
COMPASS Science Payload & Configuration

The COMPASS payload design comprises ten instruments accommodated on the spacecraft. Each instrument is based on a high-heritage representative sensor from a previous mission such as Juno, Van Allen Probes, and Europa Clipper with substantial additional shielding mass allocated to the instruments, as necessary. Section 4.2 explores potential trades that could be implemented via development and/or augmentations to the representative heritage instruments that could mitigate radiation effects without the need for such significant shielding mass. Table 3 provides the COMPASS payload mass and power table and Fig. 8 illustrates the payload configuration on the spacecraft, FoVs, location inside bays with close out. Instruments are grouped into particles, fields, and imaging suites. Next, we provide short descriptions on the various science instruments.

**Table 3:** Payload Resource Table Summary

	Mass					Power		
Instrument	#	CBE total (kg)	Addt’l Shielding (kg)	Cont.	MEV (kg)	CBE total (W)	Cont.	MEV (W)
Thermal Plasma Detector (TPD)^†^	2	14.0	0.0	10%	15.4	10.0	10%	11.0
Suprathermal Particle Detector (SPD)^††^	1	9.2	8.0	10%	19.0	9.5	15%	11.0
Energetic Particle Detector (EPD)	1	6.4	3.0	10%	10.3	3.1	15%	3.6
Relativistic Particle Detector (RPD)	1	13.4	2.7	20%	17.8	6.2	15%	7.1
Ultra-relativistic Particle Detector (UPD)	1	9.2	1.8	10%	13.3	13.2	25%	16.5
Fluxgate Magnetometer (FGM)	2	1.6^*^	0.0	10%	1.8^*^	4.2	15%	4.8
Search Coil Magnetometer (SCM)	1	7.1^*^	0.0	10%	7.9^*^	1.0	15%	1.2
Electric Field Waves (EFW)	1	13.2	0.0	10%	14.6	15.4	15%	17.7
X-Ray Imager (XRI)	1	10.0	5.0	10%	16.5	6.0	15%	6.9
E/PO Camera (EPOC)^†††^	1	3.7	2.2	10%	6.5	2.4	15%	2.7
Payload Totals		87.8	22.7		123.1	71.0		82.5

^†^Descope option: single sensor, little impact to science → trade: pitch angle vs. corotation flow coverage

^††^Descope option: remove sensor, impacts science tied mostly to particle origins (see MR1 in STM) & creates narrow energy gap of ∼30 keV between TPD and EPD for electron & proton energies

^†††^Descope option: remove completely, no impact to science

^*^Not including shared 2.6 kg boom

#### Thermal Plasma Detector (TPD)

The TPD instrument measures energy and angular distributions of thermal ion and electron plasma from ∼10 eV/Q to ∼10 keV/Q to help assess the origins, acceleration, and losses in the Jovian magnetosphere. Additionally, TPD can make mass-per-charge measurements which are critical for determining the plasma composition. The TPD sensors in the notional COMPASS payload are modeled after the PIMS instrument currently in development for the Europa Clipper mission (Grey et al. 2018; Westlake et al. 2023). The PIMS instrument, a Faraday cup design, was chosen because of its high tolerance for extreme radiation environments and significant shielding as built for Europa Clipper. Two sensors, orthogonal to each other, are implemented in the baseline payload to help ensure observability of the co-rotation vector of the magnetospheric plasma is maximized throughout the COMPASS orbit.

#### Suprathermal Particle Detector (SPD)

The SPD instrument measures the energy, angular, and compositional (mass and charge-state) distributions of suprathermal (few keV/Q to 100 s keV/Q) ions to determine the origins and acceleration processes in the Jovian magnetosphere. The SPD sensor in the notional COMPASS payload is modeled after the CHEMS instrument, an electrostatic analyzer paired with a time-of-flight subsystem, flown on the Cassini mission to Saturn (Krimigis et al. 2004) and similar instruments have been used recently, e.g., Owen et al. (2020). For COMPASS, the CHEMS instrument, which was flown in a much less severe radiation environment at Saturn, will require substantial additional shielding mass to protect its radiation-sensitive microchannel plate detectors (Table 3).

#### Energetic Charged Particle Detector (EPD)

The EPD instrument measures the energy, angular, and mass composition distributions of energetic (10 s keV to > few MeV, exact energy range is species dependent) ions and electrons to determine the acceleration and loss processes at play in the Jovian radiation environment. The EPD sensor in the notional COMPASS payload is modeled after the JEDI instruments, a time-of-flight-based design with solid-state energy detectors, flown on the Juno mission currently orbiting Jupiter (Mauk et al. 2017). For COMPASS, the JEDI instrument will only require a modest, ∼30%, increase in mass for shielding.

#### Relativistic Particle Detector (RPD)

The RPD instrument measures the energy and angular distributions of relativistic (∼1 to 10 s of MeV) ions and electrons to determine the acceleration and loss processes at play in the Jovian radiation belts. The RPD sensor in the notional COMPASS payload is modeled after the REPT instrument flown on the Van Allen Probes mission to explore the radiation belts at Earth (Baker et al. 2012). For COMPASS, the REPT instrument – a solid-state telescope with stacked SSDs – will only require modest additional shielding mass.

#### Ultra-Relativistic Particle Detector (UPD)

The UPD instrument measures the energy and angular distributions of ultra-relativistic (∼10 to 10,000 s MeV/nuc) protons & heavier ions and (∼8 MeV to > 50 MeV) electrons to make the first in-situ comprehensive measurement of the highest-energy populations in the most extreme radiation environment in the solar system. The UPD sensor in the notional COMPASS payload is a slightly modified version of the RPS instrument flown on the Van Allen Probes mission to explore the radiation belts at Earth (Mazur et al. 2012). For COMPASS, the RPS instrument – a solid-state telescope paired with a Cherenkov radiator – will require minimal additional shielding mass. An alternative and complementary design for UPD is the Pix.PAN instrument (Hulsman et al. 2023; Bergmann et al. 2024; Wu et al. 2019).

#### X-Ray Imager (XRI)

The XRI instrument measures the energy distribution of X-ray emissions (∼0.5 to 10 keV) via line-of-sight images of the Jovian radiation belts as well as precipitation into the planet’s atmosphere. The energy range is chosen to distinguish soft and hard X-rays. The XRI instrument in the notional COMPASS payload is based on a combination of the Mercury Imaging X-ray Spectrometer (MIXS; Bunce et al. 2020) and an instrument currently in development for flight on the Atmospheric Effects of Precipitation through Energetic X-rays (AEPEX) mission at Earth (Marshall et al. 2020). For COMPASS, the XRI instrument – an array of solid-state detectors with coded and pinhole apertures – will require significant additional shielding mass.

#### Fluxgate Magnetometer (FGM)

The FGM instrument measures the three-dimensional DC magnetic field, up to 128 Hz sampling, to help assess the loss processes, particle pitch angle, and plasma dynamics in the Jovian environment. The FGM instrument in the notional COMPASS payload is based on the MAG instrument flown on the MESSENGER mission to Mercury (Anderson et al. 2007). For COMPASS, the two MAG sensors – low-noise, tri-axial, fluxgate instruments – will be mounted in a “gradiometer” configuration on a single 2.6-m-long boom to ease engineering burden of magnetic cleanliness requirements. FGM requires no additional shielding mass, as the instrument electronics will be accommodated in the spacecraft’s central vault.

#### Search Coil Magnetometer (SCM)

The tri-axial SCM instrument measures the three-dimensional AC magnetic field, up to 60 kHz sampling, to help assess the wave dynamics and loss processes at play in the Jovian magnetosphere. The SCM instrument in the notional COMPASS payload is based on the search coil antenna of the WAVES instrument flying currently on the Juno mission at Jupiter (Kurth et al. 2017). For COMPASS, the tri-axial SCM sensors – high-permeability cores within a bobbin holding thousands of turns of copper wire – will be mounted on the same 2.6-m-long boom as the FGM sensors and require no additional shielding mass, as the instrument electronics will be accommodated in the spacecraft’s central vault.

#### Electric Field Waves (EFW)

The EFW instrument measures the three-dimensional AC electric field to help assess the wave dynamics and loss processes at play in the Jovian magnetosphere. EFW will be sampled up to 6 MHz to resolve the upper hybrid line for accurate plasma density determination and down to lower Perijove altitude (L < 1.02 R_J_). The EFW instrument in the notional COMPASS payload is based on the WAVES instrument flying currently on the STEREO mission observing the Sun (Bougeret et al. 2008). For COMPASS, the three EFW antennae – 6-m beryllium-copper (BeCu) stacers – will require no additional shielding mass, as the instrument electronics will be accommodated in the spacecraft’s central vault.

### Radiation Effect on Science Payload

Science instrumentation on previous Jupiter missions always struggled with low signal-to-noise (SNR) in the harshest regions of Jupiter’s magnetosphere. For example, the high intensities of very energetic charged, i.e., penetrating backgrounds, found near and inside of Europa present challenges to charged particle instruments (e.g., Kollmann et al. 2022). Therefore, SNR will be a key design driver for the COMPASS payload. Here, we perform a preliminary analysis of SNR on a few representative instruments and demonstrate methods that can be easily implemented to reduce backgrounds.

**Fig. 9:**
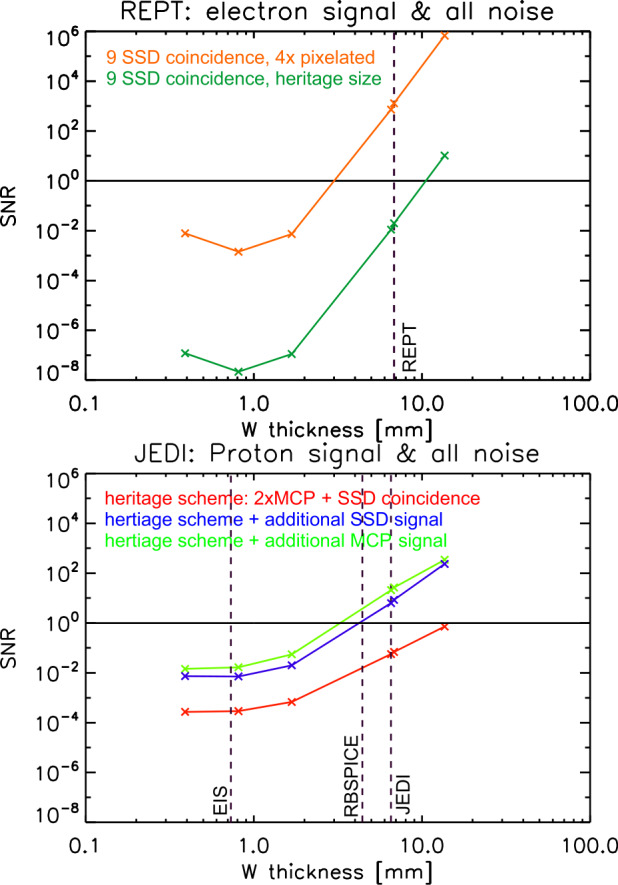
Expected signal-to-noise (SNR) ratio as a function of shielding thickness for 0.2 MeV protons measured by a Juno/JEDI-like instrument (COMPASS/EPD) and 15 MeV electrons measured by a RBSP/REPT-like instrument (COMPASS/RPD)

SNR is calculated using worst-case spectra in Jupiter’s radiation belts based on the JOSE/Salammbô physical model (Nénon et al. 2017, 2018). The signal is calculated based on the input spectrum and the nominal instrument response. To estimate the noise, we used GEANT4 to determine how the input spectra of incident protons and electrons manifest as proton, electron, and, gamma spectra behind instrument shielding using tungsten with different thicknesses. To estimate the measured backgrounds, we perform a simple forward model that includes species and energy dependent measurement efficiencies based on heritage designs. Figure 9 shows detailed SNR results for two scenarios: i) ion measurements using an EPD-like instrument and 2) electrons measurements with a RPD-like instrument.

The results in Fig. 9 illustrates there are somewhat straightforward techniques that can be implemented to increase SNR on heritage instruments without necessitating major design changes. These options can also present the basis for trade studies, e.g., mass (shielding) against complexity (adding additional coincident detectors). In general, the simplest solution is to increase the shielding mass. For example, we find that an equivalent of 6.7 mm of tungsten (W)—used by recent missions such as Juno/JEDI and RBSP/REPT—can be simply doubled to achieve a desired SNR in Jupiter’s harshest regions. An alternative to shielding is adding additional coincidence detectors into the instrument to reduce backgrounds via logic in the flight software. JEDI measures ions using a combination of two MCPs and one SSD detector. MCPs are typically used in time-of-flight based instruments for measuring timing pulses triggered by secondary electrons. Adding another MCP allows additional time pulses for redundancy and increases SNR by 3 orders of magnitude (see bottom panel in Fig. 9). REPT measures electrons using up to nine SSD detectors arranged in a stack.

The bottom panel in Fig. 9 illustrates that even this high number of coincidence detectors can be insufficient. The reason for this is that the detectors are running in saturation; that is to say, particles reach the detectors faster than they can be counted. One possible solution is to either reduce the detector size or pixelate the detectors. The latter essentially maintains sensitivity in low count environments and avoids saturation in high count environments. In summary, shielding can provide a straightforward means in reducing backgrounds, but it can add significant mass to the overall payload (see Table 3); however, other techniques such as adding additional detectors or pixelating them can significantly improve the outcome, while not growning the mass significantly. COMPASS, and other missions that want to measure extreme environments, can benefit from future research and development into background mitigation techniques.

### Flight System

**Fig. 10:**
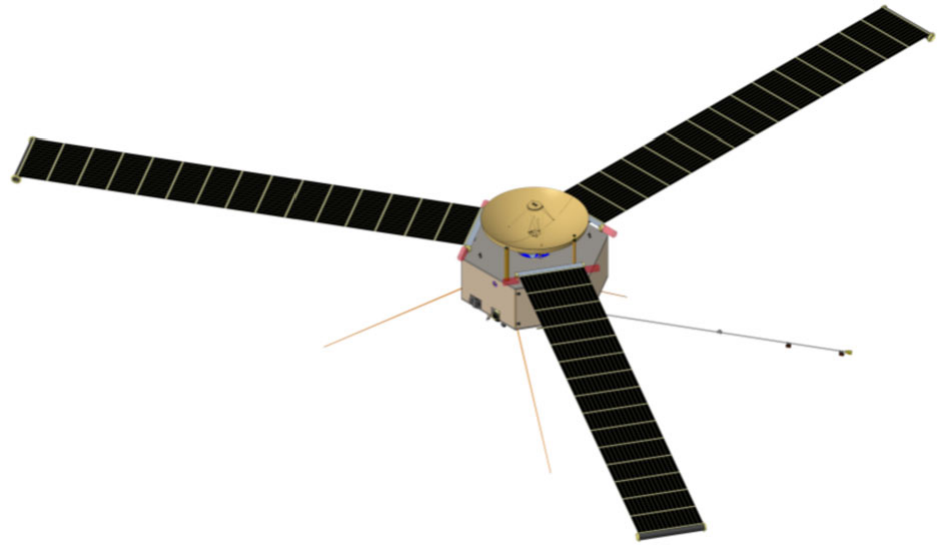
COMPASS spacecraft structure builds off heritage from IVO & IMAP. Shown is spacecraft structure with roll out solar arrays, high gain antenna shown, magnetic field boom, electric-field stacers, and bay with instruments located inside

The COMPASS flight system consists of an orbiting spacecraft and fits within the 5-m diameter of SpaceX’s Flacon Heavy Expendable fairing. No staging or other elements are required to meet the mission science objectives. All functions are incorporated on the spacecraft to meet the science objectives, including X-band communication functions with Earth, orbital maneuvers, a stable platform for the science measurements, and powering of all systems. All electronics subsystems are redundant to accommodate the 10-year mission design life. Overall, COMPASS is a spin-stabilized hexagonal spacecraft with maximum dry mass of 1456 kg. The spacecraft bus is 4.6 m across and 3.4 m high, with three Roll-Out Solar Arrays (ROSAs) mounted on three of the faces, as shown in Fig. 10.

### Spacecraft Structure

**Fig. 11:**
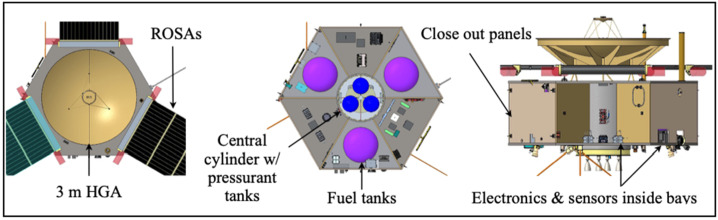
Spacecraft structure highlighting the HGA, ROSAs, fuel tanks, pressurant tanks, close out panels, and sensor arrangement inside bays

The COMPASS spacecraft will be built with an aluminum honeycomb structure, modeled on the patterns of the Io Volcano Observer (IVO) and Interstellar Mapping Probe (IMAP, McComas et al. 2018). This design baselines a hexagonal spacecraft with a central cylinder. Three fuel tanks will be located in alternating bays, with three pressurant tanks in the central cylinder. Instruments are located in the alternating three bays from the fuel tanks, with electronics boxes and other bus components spread throughout all six bays, as space permits (see Fig. 11).

All six external bays have aluminum honeycomb closeout panels. These serve two functions, providing both structural support for the bays as well as additional radiation protection for the electronics boxes, subsystems, and instruments inside. Most instruments are located just inside these closeout panels, on the top and bottom decks or on the radial panels, with small cutouts in the closeout panels for fields of view outward from the spacecraft. Instruments are not mounted directly to the closeout panels, to preserve the ability to install and remove these panels as easily as possible during I&T.

Solar arrays are modeled after the Roll Out Solar Arrays (ROSAs) recently flown on DART and the International Space Station. Three such arrays are body-mounted to the top deck, to deploy radially away from the spacecraft. The spacecraft structure is sized to be as large as possible while fitting in the 5-m SpaceX Falcon Heavy fairing, so that each structure “face” will be as large as possible, thus giving the solar arrays the maxi mum possible width.

This spacecraft will require several deployable mechanisms. Each of the three ROSAs will deploy from the spacecraft top deck, as well as a double-hinge magnetometer boom deployment from the bottom deck. All other deployments will be internal to specific instruments. Note, all deployments occur prior to arrival to the Jovian system.

### Propulsion

COMPASS will use a pressurized monopropellant hydrazine system. The hydrazine will be stored in three identical, qualified, NGIS 80451 diaphragm tanks (Fig. 11) each capable of carrying 451 kg of propellant for a total of 1456 kg. This will provide 1500 m/s of $\Delta $V to a 3230 kg launch mass. Helium pressurant will be stored in three additional composite overwrapped pressure vessels (COPV) tanks (i.e., NGIS 80436) and will allow the system to provide a constant feed pressure to the thrusters. For large $\Delta $V burns and time sensitive maneuvers, COMPASS will incorporate four 100-lbf class thrusters, notionally the Aerojet MR-104A/C. Four are needed to handle the propellant throughput required. The mission will have the option of firing a single engine or two at a time depending on the maneuver requirements. An additional four 5-lbf Aerojet MR-106E thrusters will be used for steering during large burns and twelve 1-lbf Aerojet MR-111C thrusters for ACS. Each component has flight qualified options, most of which have been flown on heritage spacecraft.

A dual-mode system was also considered for COMPASS. The use of dual mode main engines, rather than the four 100-lbf thrusters baselined, would reduce the total propellant load to 1300 kg while maintaining the same spacecraft dry mass. However, because the COMPASS structure design and launch vehicle are capable of carrying the heavier propellant load, the monoprop system was baselined. In COMPASS’s case, the monoprop system’s simplicity of design and usage, as well as significantly lower cost, wins against the additional performance provided by the more complex dual mode system. A monoprop baseline also enables the option of switching to a dual mode system to gain that added performance and reduce the propellant load if mission requirements change (increased dry mass or $\Delta $V, reduced launch vehicle capability, etc.). In addition, since the dual mode tankage would be smaller in volume, the fundamental design of the spacecraft would not need to be altered to accommodate it.

### Electrical Power

The Electrical Power Subsystem (EPS) uses a high-efficiency, peak-power-tracking, solar-array/battery architecture with significant heritage from PSP and other APL missions. Solar array (SA) power is processed by buck-topology DC/DC converters within the power system electronics (PSE) box, which regulates SA power and battery charging. The battery-dominated power bus is maintained within a voltage range of 22 to 35 V.

Three Roll-Out Solar Array (ROSA) wings provide primary power of 500 W with a total of 72 square meters of flexible blanket area. To accommodate the charged particle radiation environment, the solar cell assemblies incorporate 500 um (20 mils) coverglass. Backside shielding provided by the standard power modules that comprise the array is taken into account in the radiation degradation estimates. Radiation testing, and low-irradiance, low-temperature and room-temperature characterization and screening is baselined for the solar cells, which are optimized for this environment.

The PSE design has been flight-proven on PSP and DART, and similar slices are used on COMPASS. Four parallel buck converters process SA power. In the unlikely event of a buck converter fault, the remaining three can accommodate the load. Local, autonomous, SA electrical peak-power tracking within the PSE reduces burden on the S/C processor and improves subsystem testability. Peak-power tracking also allows all SA strings to have the same quantity of series cells, which optimizes the power available under worst-case conditions. The PSE performs constant-current, constant-voltage battery charging with default limits that can be modified by command for contingencies. Three solar array diode boxes serve as the interfaces between the SA wings and the PSE, with diode isolation of each string of cells and power bussing. The power switching unit (PSU) contains individual power services for distribution to S/C components. The PSU receives power from the PSE and provides unswitched, switched, and pulsed power services. PSU circuits have significant heritage from distribution units flown on PSP, DART, and Van Allen Probes. Individual load currents are included in telemetry. Safety busses controlled by S/C separation signals feed power to services for propulsion thrusters, RF transmission, and mechanical deployments to meet range safety requirements. A 42-amp-hour capacity lithium-ion battery supports launch and peak loads. The battery, procured from ABSL, is similar to the design flown on PSP but is larger in capacity.

### Avionics

The avionics subsystem manages the spacecraft’s command and data handling (C&DH) system. The low-power avionics, uses techniques and design approaches proven by MESSENGER, STEREO, New Horizons (NH), Van Allen Probes, and PSP, coupled with radiation mitigation strategies flown on Van Allen Probes, enable low-risk C&DH implementation. The key components of the avionics subsystem, are radiation-shielded integrated electronic modules (IEMs), distributed remote interface units (RIUs), and a radiation monitor (RadMon). The IEMs each combine C&DH and mass memory storage. The IEMs are based on the PSP modular avionics design and leverage those circuit cards to provide a high-heritage design. The SBCs provide 256 MB of SDRAM, 8 MB of MRAM, and 64 Gb of flash memory with the UT700 100 MHz processor. Housekeep data rates, as well as, the maximum record and playback rates are 1 kbps, 600 kbps, and 500 kbps, respectively. Additional PSP heritage-based components include a pair of Spacecraft Interface Cards (SCIF), two Thruster/Actuator Cards (TAC), two Instrument Interface Cards (IIF) with Solid State Recorders (SSR), and two DC/DC converters. Two strings of Remote Interface Units (RIUs) provide a total of 120 analog channels for temperature sensing. The engineering RadMon is an APL-designed radiation monitor for Europa Clipper, and it will monitor total dose and dielectric charging in real time. In addition, RadMon benefits the mission by assessing the radiation health of the spacecraft.

### Guidance & Control

COMPASS is predominately a passive spin-stabilized spacecraft, drawing inspiration from IVO and Juno. Nominally, the spacecraft maintains a constant spin about its fixed antenna boresight axis at a rate of 2 rotations per minute (RPM), keeping its antenna pointed toward Earth for telecommunications. This spin motion also allows for spacecraft to sweep its instrument suite to get a complete view of its environment, which is a top-level mission requirement. The spin rate was chosen to provide stability while keeping propellant usage during precession maneuvers at an acceptable level, while providing a suitable scan rate to the instruments. This passive mode will be routinely perturbed via thruster firings to precess the spin axis to maintain line of sight communications to Earth. On the few occasions where large modifications to the trajectory are required (Deep Space Maneuver (DSM), Jupiter Orbit Insertion (JOI), Perijove Raise Maneuver (PRM)), the spacecraft will precess the spin axis to align its main $\Delta $V thrusters in the direction of the required thrust vector, and perform the burn maneuver while spinning for stability. For smaller Trajectory Correction Maneuvers (TCMs), the spacecraft may choose to maintain its Earth-pointing posture and pulse the smaller thrusters to achieve the desired correction to minimize propellant consumption. Thruster firings will excite spacecraft nutation and solar array motions that will dampen out over time, accelerated by two nutation dampers.

Due to its spinning nature, COMPASS does not require continuous active attitude control. However, providing better than 0.25° attitude knowledge for the instruments requires a sufficient level of sensing. This is achieved through a pair, for redundancy, of Sodern Hydra TC Star Trackers and an internally redundant Northrop Grumman Scalable Space Inertial Reference Unit (SSIRU), containing four gyros and four accelerometers. The star trackers are mounted with boresights 15° off the spin axis to reduce the perceived rotational rate to ensure a robust star lock and thus would provide 6 arcsecond accuracy to their boresights and 50 arcsecond accuracy about the boresight (3$\delta $). The SSIRU allows for closed loop trajectory adjustments as well as provides rate information that can be integrated to provide attitude information for situations where the star trackers are physically obstructed or momentarily affected by radiation. Two Sun Sensors provide additional position information relative to the Sun and are primarily used for safe mode; however, precession maneuvers and TCMs may utilize the Sun pulse from these sensors to properly phase thruster firings if it is determined that the on-board attitude knowledge is degraded and the Sun vector is separated from the spin axis. This thruster control method using Sun Sensors has been used many times on-orbit, including the Van Allen Probes and planned for IMAP. All components were chosen for the purpose of establishing the baseline subsystem design.

### Communications

**Fig. 12:**
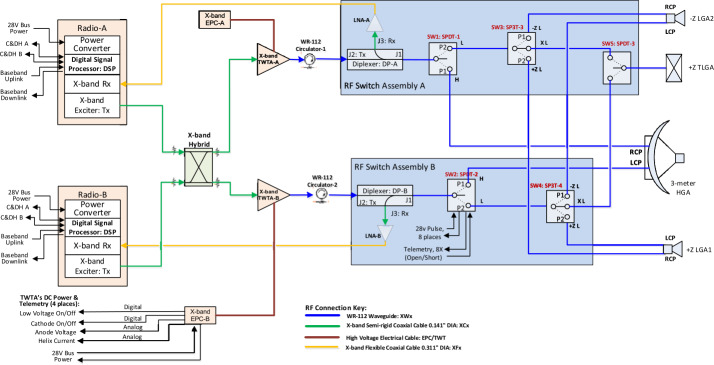
COMPASS telecommunications subsystem

The telecommunications subsystem (Fig. 12) characteristics are driven by the data volume required during the shortest Science Phase 2 orbit durations, down to 16.7 days. The most prominent result of this is the 3-m HGA, mounted on the aft of this spinning spacecraft. Science data downlinking at X-band from a 65-W TWTA for 8 hours a day, 5 days a week is sufficient to complete the transfer of 1.37 GB of compressed data plus a 50% margin at Jupiter’s maximum Earth range of 6.45 AU. All communication is through NASA’s Deep Space Network (DSN). The science data downlinks require use of a single, 34-m DSN station and HGA pointing accuracy maintained to within ±0.4 degrees. Use of the 70-m DSN station will increase our downlink allocation and enhance science return. Emergency operations at Jupiter range would require a 70-m DSN station if pointing cannot be maintained.

Key trades defined the telecommunications subsystem. First, NASA directs all new missions to baseline Ka-band downlinks, and indeed that does inherently offer more gain. However, it also adds mass and complexity and, more critically, a pointing accuracy requirement of ±0.1 degree or better which is not feasible for this spin-stabilized spacecraft. Second, as the spacecraft is more power constrained than mass constrained, the 3-m HGA was selected to minimize the TWTAs’ demand for larger solar arrays.

Two opposing (fore and aft) low-gain antennas (LGAs) and a toroidal low-gain antenna (TLGA) compliment the HGA to provide coverage for all mission phases. During launch and early operations (LEOP) and the Earth gravity assist (EGA) maneuver, the LGAs are sufficient to support downlink throughput of up to 1 Mbps. A deep-space maneuver at approximately 4.3 AU Earth range is covered by the TLGA (which provides a donut-shaped pattern perpendicular to the spin axis) as the Earth is visible at an angle 90 degrees off the spin axis. During this maneuver, only minimal data rates of 7.8 bps for uplink and 10 bps for downlink are supported. During Jupiter Orbit Insertion (JOI) and Perijove Raise Maneuver (PRM), the spacecraft is off-pointed by 50 degrees and command and telemetry links cannot close with sufficient margin, however, beacon tones are still available to indicate status.

Telemetry, tracking, and control (TT&C) is provided through redundant APL Frontier Radios. A next-generation version is under development to replace the current “Classic” version and would be available by the time COMPASS is underway. The Frontier Radio Classic has significant flight heritage on NASA’s Van Allen Probes, Parker Solar Probe, and Double Asteroid Redirection Test (DART) missions as well as the United Arab Emirates’ Hope Mars mission, and by the time COMPASS would launch, NASA’s Europa Clipper and Dragonfly missions. The next-generation version will employ major reuse of the software-defined radio (SDR) algorithms and processing while taking advantage of more advanced modern hardware.

### Mass & Power Resource Table

Table 4 depicts the mass and power resource table for the COMPASS mission concept.

**Table 4:** COMPASS mass and power resource table

	Mass		Average Power	
	CBE (kg)	MEV (kg)	CBE (W)	MEV (W)
Structures & Mechanisms	352.66	402.93	-	-
Thermal Control	31.20	34.37	85.00	97.75
Propulsion (Dry Mass)	276.54	290.37	-	-
Attitude Control	45.38	48.49	41.60	43.68
Command & Data Handling	35.12	38.15	55.15	63.11
Telecommunications	66.00	74.67	124.50	137.43
Power	293.26	336.09	41.40	46.31
Harness	102.92	108.06	12.56	14.12
Science Payload	87.8	123.1	71	82.5
Total Flight Element Dry Bus Mass	1290.88	1456.23	431.21	484.90
Propellant Mass	-	1630	Contingency: 13% Margin: 142% Tot. Margin: 167%	
LV Capability	-	5160		

### Mission Design

The COMPASS trajectory design is composed of three mission phases: launch and interplanetary cruise; capture into the Jovian system; and a multiphase science tour (see Table 5).

**Table 5:** Mission phases, with assumptions and the major events

Mission Phase	Description
I. Launch & Interplanetary Cruise	• Launch, Falcon Heavy Expendable • Deep Space Maneuver (DSM) • Earth Gravity Assist (EGA) • Jupiter system arrival
II. Capture into the Jovian System	• Io-flyby (I1) • Jupiter Orbit Insertion (JOI) • Perijove-Raise Maneuver (PRM) • Io-flyby (I2)
III. Science Tour	• Science Phase I (high-inclination, larger PJ) • Science Phase II (low-inclination, lower PJ) • Disposal via Jupiter impact

The goal of mission phases I and II is to deliver COMPASS to an orbit that meets the requirements for Science Phase I, while setting up conditions for efficient transition into Science Phase II. The science campaigns/phases can be further broken into their respective requirements flowed down from the science and measurement objectives. Here, $r_{p} $and $r_{a}$ represent perijove and apojove radii, respectively, $i$ represents inclination relative to the Jovian equator, and local solar time is denoted as LST. The radius of Jupiter is defined as R_J_ = 71,492 km. Previous concepts to study the Jovian magnetosphere and radiation environment are structured such that the initial science orbit lies in the Jovian moon plane, and inclination is increased via flybys of Callisto (Campagnola & Kawakatsu 2012). In the COMPASS study, this paradigm is reversed so that the tour is initially inclined, with Io flybys executed on each revolution of the spacecraft about Jupiter to reduce orbital period and apojove radius. Then, while still in this inclined orbit, a transfer to Callisto is performed, and a series of Callisto flybys enable simultaneous reduction of both inclination and perijove radius, thus covering lower inclinations in the last phase of the mission. To reduce radiation Total Ionizing Dose (TID) and risk of Europa impact, non-zero inclination is maintained during the entire Science Phase. The first phase has the following characteristics: i) orbital inclination relative to Jovian equator i ≥ 30°; ii) perijove within 4 R_J_ $\leq \ r_{p}\ \leq $ 6 R_J_, and within dusk quadrant 15:00 ≤ LST ≤ 21:00 hrs; and iii) apojove within 30 R_J_ $\leq \ r_{a}\ \leq $ 90 R_J_, and within dawn quadrant 3:00 ≤ LST ≤ 9:00 hrs. The second science phase can be summarized as: i) orbital inclination relative to Jovian equator $i\ \leq $ 20°; ii) perijove within 1 R_J_ $\leq \ r_{p}\ \leq $ 2 R_J_, and within dusk quadrant 15:00 ≤ LST ≤ 21:00 hrs; iii) provide coverage out to r = 30 R_J;_ iv) ≥ 3 orbits; and v) ensure safe disposal, given possible spacecraft failure on any orbit in this phase.

#### Launch and Interplanetary Cruise

**Fig. 13:**
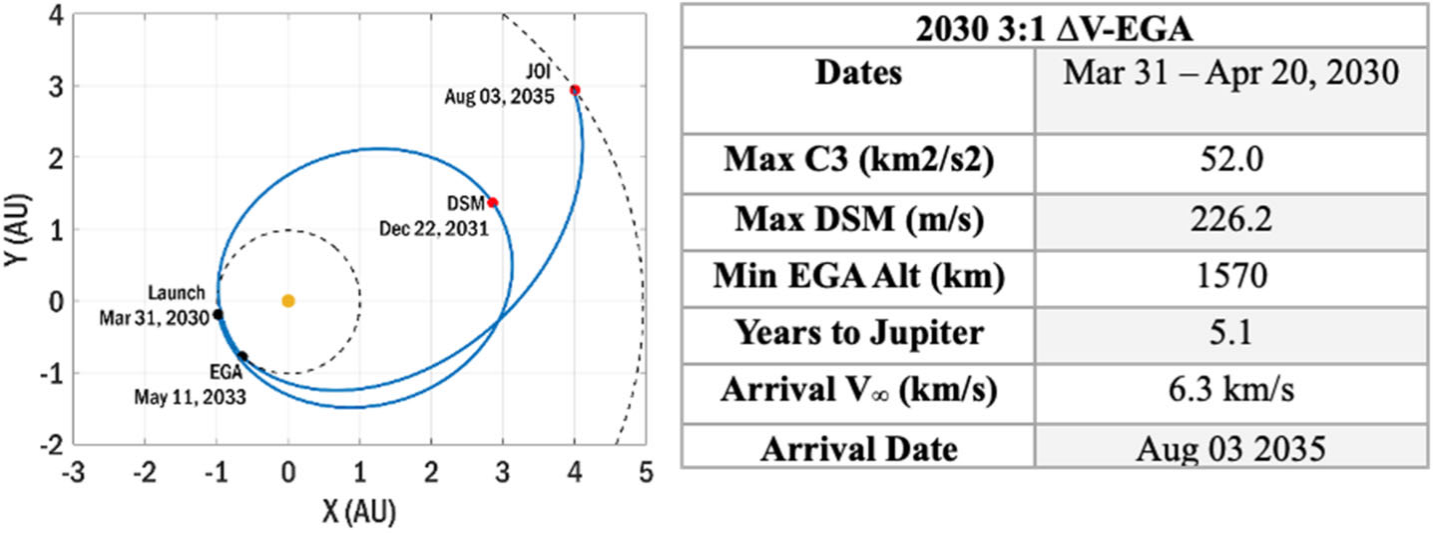
Interplanetary Cruise to Jupiter on a 3:1 $\Delta $V-EGA

Assuming the Falcon Heavy Expendable, a 3:1 $\Delta $V-EGA cruise trajectory is enabled. Here, a higher launch C3 is achievable, injecting the spacecraft into a roughly 3:1 resonance with Earth. A DSM at aphelion targets an increased $V_{\infty} $ at the EGA, enabling transfer to Jupiter. During the EGA, COMPASS’s payload will be turned on to operate the instruments for science and cross calibration opportunities in Earth’s relatively observatory dense magnetosphere. Launch in 2030 is assumed for this point design, however the flight system design is scaled to meet the maximum propellant needs expected for any launch from 2030 – 2042. Launch declination is constrained ≤ 28.5° for all solutions. For each day in the launch period, Jupiter arrival is constrained to a single epoch to enable the design of a single capture sequence and science tour. The date of arrival to the Jovian system is initially selected to minimize the DSM+JOI $\Delta $V, and is then adjusted forward ∼16 days to optimize moon transfer phasing during the science tour. A summary of the 2030 launch appears in Fig. 13. Details on the launch and interplanetary trade space are provided in the mission

#### Capture into the Jovian System

Upon arrival to the Jovian system, a capture sequence inserts the spacecraft into Jovian orbit. The capture sequence that best aligns with the goals of the science campaign is an Io-aided (I1) JOI maneuver, followed by a PRM at apojove to counteract solar gravity perturbations and retarget a second Io flyby (I2). All Io flybys are modeled at 300 km altitude, and JOI and PRM are 871.7 m/s and 22.3 m/s, respectively. Because the I1 flyby occurs after perijove, it is navigationally risky to execute JOI at perijove (prior to I1). For this reason, JOI is delayed to 1-hour after exit from the I1 sphere-of-influence. To place perijove over Jupiter’s northern hemisphere, the Io flybys are targeted at the orbit descending node. This improves detection of particle losses in regions where Jupiter’s magnetic field changes more steeply as a function of latitude and longitude, and enables the X-ray imager to observe Jupiter’s northern main aurora and atmosphere.

#### Science Tour

The COMPASS science tour is composed of two mission design phases that are tailored to meet the requirements for the two science phases. They first mission phase is Io pump-down phase that consists of an inclined orbit (i ≥ 30°) that uses repeated flybys of Io to reduce orbit period and apojove, a transition to 1:1 resonance with Callisto, and intersection of ascending node with Callisto’s orbit and a finally a transfer to Callisto to begin the second science phase. The second mission phase is termed the Callisto crank-down phase because it uses repeated flybys of Callisto to simultaneously reduce inclination and perijove radius and inclination to r$_{\mathrm{p}}\ \leq $ 2 R_J_ and i ≤ 20°, required for the second science phase.

**Fig. 14:**
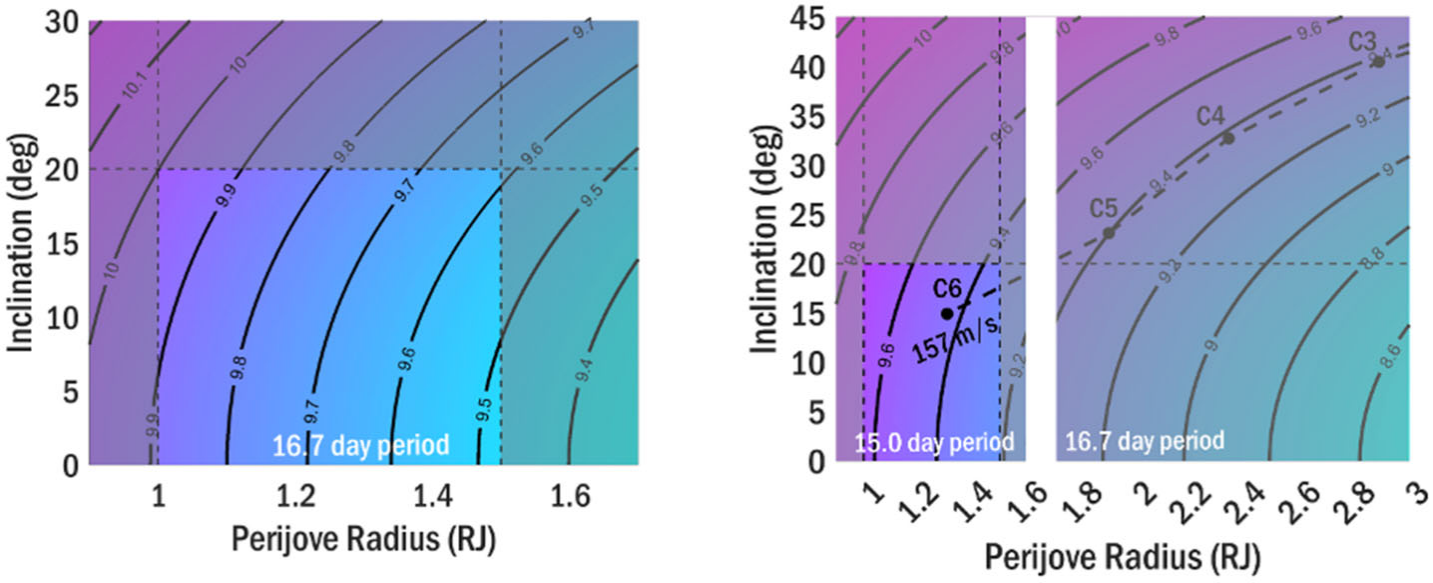
Callisto flybys are used to simultaneously reduce perijove radius and inclination. Colormaps with curves of constant $V_{\infty} $ (km/s) overlaid in black)

To transfer between Io and Callisto from an inclined orbit, both orbit node crossings must intersect each of the moon orbits, leading to a fairly constrained geometry. The benefit of targeting such a condition is that the entire science tour can remain inclined, reducing TID and risk of Europa impact. By design, COMPASS’s orbital tour ensures no intersections with Europa’s orbit, minimizing any chance of collision with Europa prior to entry into Jovian atmosphere at mission end-of-life. Figure 14 shows the targeted orbital element space for Science Phase II, i.e., the Callisto flyby conditions that enable $r_{p}\ \leq $ 1.5 R_J_ with $i\ \leq $ 20. In Fig. 14 (left panel), curves of constant $V_{\infty} $ are plotted in $r_{p}$ - $i$ orbital element space, assuming 1:1 resonance with Callisto and with regions that violate the second science phase conditions grayed out. The result is a targeted range of Callisto $V_{\infty} $ magnitudes from ∼9.5 - 10 km/s. With the inclusion of Jupiter gravity harmonics, specifically J2, the $\Delta $V to continue targeting subsequent Callisto flybys increases significantly, especially as $r_{p}$ and $i$ decrease. By allowing the orbital period to reduce from the 1:1 resonance after a final Callisto flyby (C6 for the tour presented here), a lower perijove can be achieved for reduced Callisto $V_{\infty} $ and fewer Callisto flybys. The path of the final COMPASS science tour appears in Fig. 14 (right panel), with joined maps of $r_{p}$ - $i$ space for the 1:1 resonance, and the post-C6 orbital period of 15 days. The outgoing C6 inclination is constrained ≥ 15° to reduce TID, and the final tour is optimized from launch through end of Science Phase II in a high-fidelity model, including solar gravity, Jupiter J2, and Io, Europa, Ganymede and Callisto point-mass gravity while inside Jupiter’s sphere-of-influence.

**Fig. 15:**
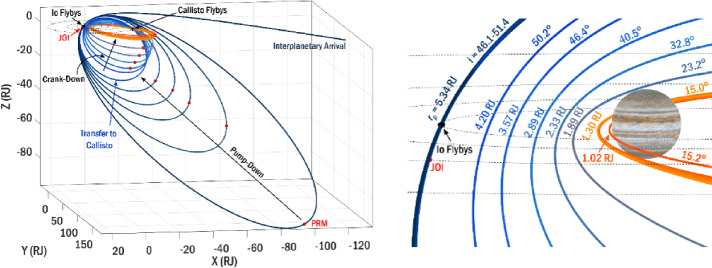
COMPASS science tour, flybys, and maneuvers appear as black and red points, respectively. Blue curves represent Science Phase I, orange curves represent Science Phase II

**Fig. 16:**
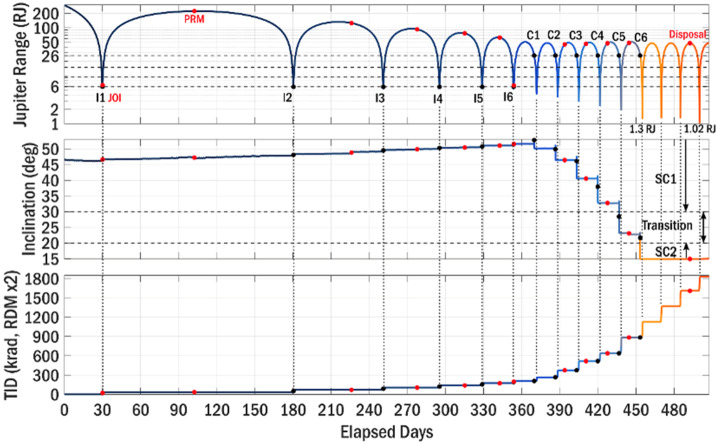
Time history (beginning 30 days prior to I1) of Jupiter range, inclination relative to the Jovian equator, and total ionizing dose over the science tour; perijove passes connected by vertical lines

The final tour associated with the path in Fig. 14 is plotted in the Ecliptic-J2000 frame in Fig. 15. Figure 15 (left panel) shows the full tour from interplanetary arrival through disposal, and Fig. 15 (right panel) focuses on the changes in perijove radius and inclination during the Callisto crank-down phase. A summary figure showing the evolution of perijove, inclination, and TID appears in Fig. 16. Note that from flybys C5 to C6, both perijove and inclination are in between the required values for Science Phases I & II. This period is defined as a “Transition” between the two science campaigns, but valid science is still contributed during this time.

#### Disposal

After completion of the first 3 orbits associated with the second science phase campaign, a 150 m/s disposal maneuver is performed at apojove to reduce perijove to 1.022 R_J_, enabling Jupiter J3 gravity perturbations to further reduce perijove until “impact” with Jupiter occurs 66 days later. Here, impact is defined as spacecraft vaporization due to Jovian atmospheric entry. While perturbations from J2 will drag the orientation of the orbit, it would not cross Europa’s orbit until 74 days after impact. This disposal strategy enables an extended mission without requiring s/c survival to ensure impact. The time to impact can be adjusted by changing the magnitude and/or date of the disposal maneuver. The maneuver could also be delayed or executed earlier, depending on radiation degradation assessments during the tour.

#### $\Delta $V Budget

The $\Delta $V budget for the COMPASS trajectory is provided in Table 6, and covers any launch year given the assumptions listed. Both deterministic and statistical $\Delta $V are included in the allocated budget, and an additional 2% margin for unallocated $\Delta $V is assumed.

**Table 6:** COMPASS $\Delta $V budget

Maneuver Name	ΔV (m/s)		Assumptions
	Deterministic	Statistical	
Launch cleanup	-	20	C3 ≤ 52 km^2^/s^2^
DSM (3:1 ΔV-EGA)	230	5	3:1 ΔV-EGA
EGA	-	10	Flyby targeting + cleanup
JOI	875	25	Io-aided JOI
Tour	157	125	Flyby targeting and cleanup
		5	3% of tour deterministic
Disposal	150	-	Targeting perijove at 1.02 R_J_
Subtotal	1412	190	Total allocated ΔV
Unallocated Margin	28		2% of deterministic total

### Concept of Operations

The trajectory elements and critical events are similar to previous missions operated at APL, and the mission operations can be supported using existing APL Mission Operations Center (MOC) infrastructure and NASA Deep Space Network (DSN) capabilities. Post-launch commissioning is expected to take approximately five weeks, during which there is near-continuous DSN coverage in the first week, gradually reducing to a single 8-hour X-band communications pass per day by the end of the period. The cruise phase is 5.5 years, including a deep space maneuver (DSM) and an Earth gravity assist (EGA). The spacecraft will operate in spin-stabilized mode, with annual checkout activities. Instruments will be on during early cruise instrument checkouts, around the EGA, and for annual checkouts during cruise. Operating the instruments in continuous high-resolution mode (in-situ payloads) and special “burst” campaigns (remote sensing payloads) during the EGA will be particularly advantageous for checkout, in-flight calibrations, and comparisons to other observatories in the well-observed Terrestrial magnetosphere system; EGA observations may even provide publishable scientific results in collaboration with additional NASA and other observatories at Earth. During most of cruise, DSN 34-m antennas will be utilized for three 8-hour X-band communications passes per week to conduct typical operations including uplink of command loads every 3-4 weeks, regular downlink of spacecraft engineering data, and real-time evaluation of spacecraft health and safety. DSN coverage is increased before and after the DSM and EGA to support ranging and navigation, as well as instrument data downlink after EGA.

Jupiter approach begins approximately five months before Jupiter orbit insertion (JOI). During Jupiter approach, instruments will be on in survey mode for science characterization of the solar wind. DSN coverage increases in this period compared to cruise, to support ranging and navigation approaching JOI. Given the X-band downlink data rates available using the high-gain antenna at this time, and a cadence of three 8-hour DSN communications passes per week, science data collected can be downlinked within the same week, so there are no concerns about onboard data buildup prior to JOI and Science Phase operations. Continuous critical event DSN coverage will be required for ten hours around the JOI burn. Instruments will be turned off during the JOI burn, but turned on again soon after, providing the “first-light” observations from COMPASS within the inner Jovian magnetosphere. A few months after JOI, there is a large periapsis raise maneuver (PRM), with increased DSN coverage before and after to support ranging and navigation.

**Fig. 17:**
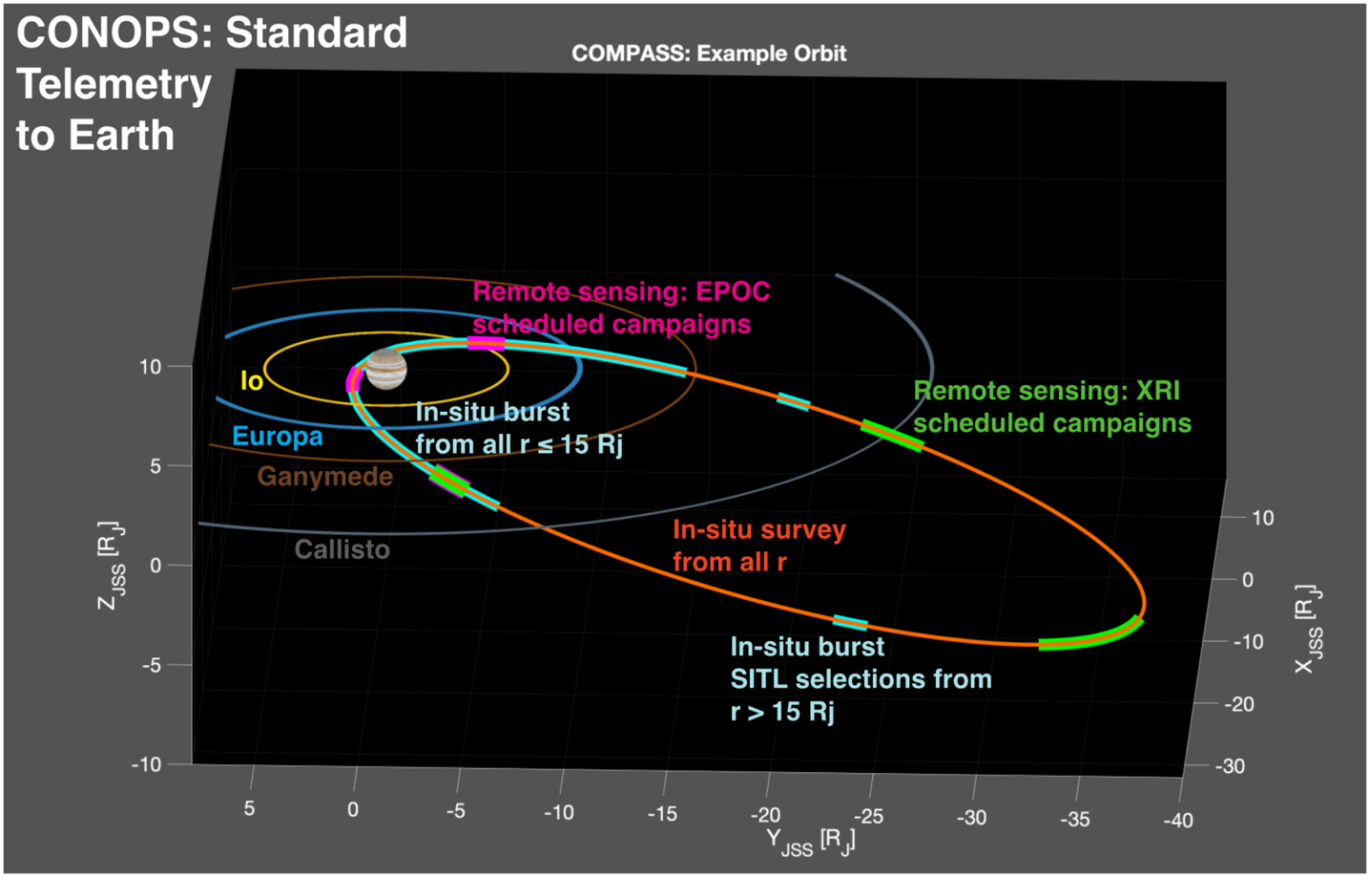
COMPASS concept of operations plan for a Science Phase II

Science Phase I begins soon after the PRM and includes thirteen orbits of Jupiter over the 1.5-year baseline. The Science Phase is roughly divided into Phase 1 (30° or higher inclination) and Phase II (low inclination, $r_{p}$ between 1 and 2 R_J_) orbits. During both phases, the communications cadence is approximately five 8-hour X-band pass per week (seven days) with a single 34-m DSN antenna. Command loads are uplinked once every two weeks. In order to maximize the value of the science return despite limitations on downlink capability from Jupiter, the science data collection approach is to maximize onboard collection, and then downlink only selected portions of the high-rate (“burst mode”) data. During every orbit of the Prime Science Phases (see Fig. 17), COMPASS acquires and records (onboard) data from all of the in-situ payloads (particles: TPD, SPD, EPD, RPD, and UPD; fields: FGM; and duty-cycled waves data: FGM, SCM, and EFS) in *both* survey and burst modes. Remote sensing (XRI and EPOC) payloads are operated during pre-scheduled image-capture campaigns for moons and Jupiter (XRI and EPOC) and radiation belts (XRI) based on relative orbit, target, and FoV orientations. The remote sensing payloads data acquisition modes and rates are summarized in the Appendix. The standard products that will be downlinked to ground from every orbit during the Prime Science Phases include: all in-situ burst data from r ≤ 15 R_J_; all survey data from the entire orbit; all remote sensing data from the scheduled acquisition campaigns. Using the survey data from all locations at r > 15 R_J_, ground-in-the-loop decisions made by the science team upon review of the downlinked survey data (i.e., “scientist-in-the-loop” or “tohban” review and prioritization) will inform data management decisions for the collected high-rate (burst) science data; the telemetry budget accounts for additional burst-rate telemetry to be downlinked to Earth from selected periods of high-interest from r > 15 R_J_. The onboard data storage capacity allows for storage of approximately 1.5 months’ worth of data to account for this approach, as well as any anomalies, thus ensuring that these data management decisions will not be particularly time-critical for the mission and science operations teams.

During Phase I and Phase II orbits, most of the in-situ instruments collect data continuously at both survey and burst rates throughout the orbit. The burst-rate particle data ensures adequate sampling throughout every COMPASS orbit of the particle distributions required to close on COMPASS science (see the STM). For example, during the fastest portions of COMPASS’ orbital tour, the observatory crosses L-shells at a maximum rate during perijove passes of $\Delta $L/$\Delta $t = 0.02 L-shells/minute, and in burst-mode (standard inside of r ≤ 15 R_J_ and thus available during *all* perijove passes), particle distributions (including phase space densities) will be available every 15-seconds (i.e., every 1/2 -spin or $\Delta $L ≥ 0.005). This is also true for the wave spectral data, which will be collected at 6-samples per second in burst mode. As alluded to above, due to the large data volume they generate, the in-situ SCM and EFW instruments cannot capture data at burst rates throughout the orbit and must be duty cycled. The remote sensing instruments acquire data at lower survey rates throughout the orbit, with selected imaging periods during pre-scheduled image-capture campaigns. These include XRI imaging campaigns targeting Jupiter, moons, and the radiation belts, and EPOC imaging campaigns for the moons and Jupiter. Reiterating: All of the survey rate data from the instruments and the imaging campaign data are downlinked, along with a subset of collected burst data – data from within 15 R_J_ and selected burst data from the rest of the orbit, based on ground-in-the-loop selection by the science team. With this strategy, the mission science data return from the Prime Science Phase is 230 Gb, including 174 Gb from Science Phase I, and 56 Gb from Science Phase II orbits, with 25% or greater margin compared to available downlink capability in each orbit.

The total mission science data return is 300 Gb. In total, 15,620 hours of DSN time over 1816 passes is needed to support the entire mission. This data acquisition and downlinked telemetry strategy is fully intended to keep COMPASS science operations simple and manageable while simultaneously optimizing data return and availability for state-of-the-art and unprecedented studies of Jupiter’s magnetosphere and radiation environment. Such a “scientist-in-the-loop” approach is novel for Jovian science missions, and it should enable unexpected, discovery-level science above and beyond of the primary science goals. Furthermore, this standard data acquisition and downlink approach and dedicated system resources to ensure the sheer magnitude of burst-rate data from all COMPASS payloads offers unprecedented levels of high-quality data from the Jovian system and ensures discovery-level science during both Prime Science Phases and closure on COMPASS science goals and objectives.

## Mission Life-Cycle Cost

The COMPASS mission is of Concept Maturity Level 4 (e.g., Wheatcraft and Lewis 2018). The payload and spacecraft estimates capture the resources required for a preferred point design and take into account subsystem level mass, power, and risk. Our estimate also takes into account the technical and performance characteristics of components. Estimates for Science, Mission Operations, and Ground Data System elements whose costs are primarily determined by labor consider the Phase A–D schedule and Phase E timeline.

The result is a mission estimate that is comprehensive and representative of expenditures that might be expected if the COMPASS mission is executed as described. The COMPASS Phase A–F mission cost, including unencumbered reserves of 50% (A–D) and 25% (E–F), is ∼ $1.2B in fiscal year 2022 dollars (FY$22), as shown in Table 7. COMPASS was selected for the Solar and Space Physics Decadal Survey (NASEM 2025) Technical Risk and Cost Evaluation (TRACE) process. The TRACE process estimated the total mission cost of COMPASS to be ∼$1.5B FY24. That estimate includes pre-Phase A through Phase F and excludes the launch vehicle and threats. TRACE evaluated COMPASS as having a “medium” risk.

**Table 7:** Estimated Phases A-F COMPASS mission cost by WBS elements

COMPASS Mission ROM Estimate (FY22$M)					
WBS	Description	Ph A-D	Ph E-F	Total	Notes
	Phase A	$6.0	-	$6.0	Assumption based on previous studies
1/2/3	PM/SE/MA	$78.1		$78.1	A-D: Wrap factor based on recent NFs and APL missions E-F: Bookkept with WBS7
4	Science	$21.8	$26.4	$48.2	Cost per month of recent NFs and APL missions
5	Payload	$134.5	-	$134.5	Parametric and analogy based estimates
6	Spacecraft	$262.9	-	$262.9	Estimated via parametric models
7	Mission Ops	$24.1	$33.2	$57.3	Cost per month of recent NFs and APL missions
8	LV	$210	-	$210	Falcon Heavy Expendable Placeholder
9	Ground Data Systems	$11.3	$1.8	$13.1	COMPASS specific estimate from GDS lead
10	I&T	$61.7	-	$61.7	APL historic I&T % of HW (incl. testbeds)
	Subtotal	$810.5	$61.3	$871.8	
	Reserves	$297.3	$15.3	$312.6	50% B-D, 25% E-F, excludes LV
	Total w/ reserves	$1107.8	$76.6	$1184.4	
	Total w/o LV	$897.8	$76.6	$974.4	

### Mission Ground Rules and Assumptions

The ground rules and assumptions used for estimating the mission life-cycle cost of COMPASS are: i) mission costs are reported using the level-2 (and level-3 where appropriate) work breakdown structure (WBS) provided in NPR 7120.5E; ii) cost estimates are reported in fiscal year 2022 (FY22) dollars; iii) the NASA New Start inflation index provided by the Planetary Mission Concept Studies Headquarters (PMCS HQ) was used to adjust historical cost, price data, and parametric results to FY22 dollars if necessary; iv) the mission does not require Technology Development dollars to advance components to TRL 6 because all COMPASS mission components are TRL 6 or greater; v) a launch vehicle cost estimate of $210M is held in WBS 8 and assumes a SpaceX Falcon Heavy Expendable launch vehicle; and vi) Phase A–D cost reserves are calculated as 50% of the estimated costs of all components excluding the launch vehicle. Phase E–F cost reserves are calculated as 25% of the estimated costs of all Phase E elements.

### Cost Benchmarking

**Fig. 18:**
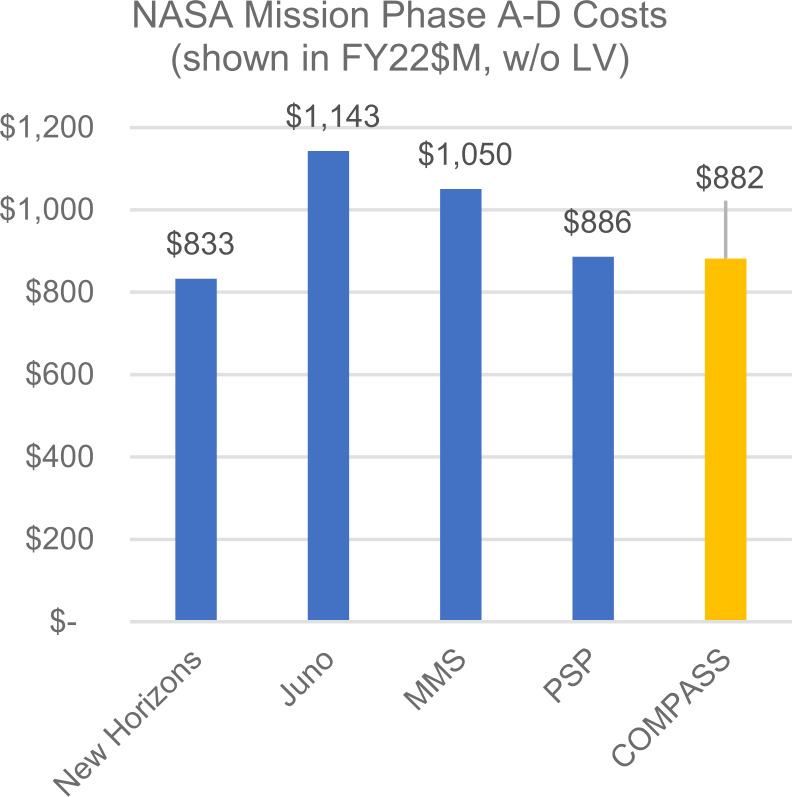
Phase A-D cost with comparable NASA missions

The cost and scope of the COMPASS concept corresponds well with the NASA missions shown Fig. 18. The estimated cost to develop COMPASS compares favorably to these NASA missions with an average cost of $970M. Excluding LV, the Phase A-D COMPASS estimate of $882M FY22$ would put it in the a Planetary New Frontiers mission class or Heliophysics Solar Terrestrial Probe cost range.

### Methodology & Basis of Estimate

The COMPASS CML 4 mission cost estimate is a combination of high-level parametric and analog techniques and incorporates a wide range of uncertainty in the estimating process. No adjustments were made to remove the historical cost of manifested risk from the heritage data underlying the baseline estimate. Therefore, before reserves are applied, the estimated costs already include a historical average of the cost of risk. This approach is appropriate for capturing risk and uncertainty commensurate with early formulation stages of a mission. The following describes the basis of estimate for each element.

#### WBS 1, 2, 3 Project Management, Systems Engineering, Mission Assurance (PM/SE/MA)

Since these WBS functions depend on multiple mission- and organization-specific characteristics (Hahn 2014), cost analogies to comparable historical missions are preferred over cost model output, which does not take the mission into account. Existing analyses demonstrate that hardware costs are a reliable predictor of these critical mission function costs. APL has conducted thorough and rigorous analyses of PM/SE/MA costs, both for historical APL missions and for analogous missions. The PM/SE/MA estimate for COMPASS relies on APL’s analysis of historical PM, SE, and MA practices on Van Allen Probes, Parker Solar Probe (PSP), and New Horizons (NH). Van Allen Probes and PSP in particular include costs associated with current NASA requirements (e.g., Earned Value Management System (EVMS), NASA 7120.5F). COMPASS’s total mission PM/SE/MA cost is 15.9% of the flight system (payload + spacecraft + I&T). This percentage is allowed to vary along with hardware costs as part of the mission cost risk analysis, discussed below, to capture uncertainty (particularly given CML 4-level design phase).

#### WBS 4 Science

This element covers the managing, directing, and controlling of the science investigation. It includes the costs of the Principal Investigator (PI), Project Scientist (PS), science team members, and activities. The Phase A–D and E–F science estimate is an analogous estimate based on the cost per month of NH, MESSENGER, Cassini, Dragonfly, OSIRIS-Rex, and Juno. NH is APL’s most recently-flown New Frontiers mission and MESSENGER is a recent historical data point for planetary orbital science. The analogy costs are representative of expenditures for science on a typical New Frontiers mission. The estimate reflects the manpower needed to create various data products as well as to ensure closure to science objectives.

#### WBS 5 Payload

The WBS 5 estimate includes a science payload of 10 instruments and payload-level PM/SE/MA (Table 8). The 8.2% cost-to-cost factor for estimating payload PM/SE/MA costs is based on the Van Allen Probes, NH, MESSENGER, and PSP payload suite cost data with PM/SE/MA costs estimated as a percentage of the payload hardware. Technical management and systems engineering costs for individual instruments are carried in their respective instrument development costs. Given the early design phase, multiple approaches are used to estimate each instrument to capture the potential range in cost. This includes two parametric estimates that rely on different sets of input variables (SEER Space and NICM 9). An average of the two parametric estimates is used as the point estimate to prevent estimate bias (high or low). These estimates are subject to a cost risk analysis (discussed below) to further quantify uncertainty. No technology development is required for the payload.

**Table 8:** COMPASS WBS 5 costs in FY$22M

COMPASS Mission Estimate (FY22$M)			
WBS	Description	Total	Notes
5	Payload	$130.2	Parametric and analogy-based estimates
5.1	PL PM/SE/MA	$9.9	Based on NH, PSP, Van Allen Probes, MESSENGER
5.2	Thermal Plasma Detector (TPD)	$12.6	Average of NICM 9/SEER Space Estimates
5.3	Surprathermal Particle Detector (SPD)	$15.5	
5.4	Energetic Particle Detector (EPD)	$12.0	
5.5	Relativistic Particle Detector (RPD)	$13.4	
5.6	Ultra-relativistic particle detector (UPD)	$17.5	
5.7	X-Ray Imager (XRI)	$21.2	
5.8	Flux Gate Magnetometer (FGM) incl. boom	$8.0	
5.9	Search Coil Magnetometer (SCM)	$2.8	
5.A	Electric Field Waves (EFW)	$11.2	
5.B	Education/Public Outreach Camera (EPOC)	$6.1	

#### WBS 6 Spacecraft

The WBS 6 estimate includes the spacecraft (SC) bus, flight software, component engineering, and radiation shielding (Table 9). SC PM/SE/MA is carried in WBS 1, 2, and 3 consistent with APL in-house builds [Hahn 2015]. The basis of estimate relies primarily on parametric models. The exception to this is the propulsion system, estimated via a ROM by a propulsion subject-matter expert. An average of two parametric estimates is used as the point estimate to mitigate estimate bias (high or low). SEER Space is one of the primary estimating methodologies because it was designed specifically for missions in early formulation stages. TruePlanning is also utilized as it provides a cost estimate at the component level. No technology development is required for the SC. The two parametric estimates are within 15% of each other (which is a reasonable range given different input variables). Cross-checks are shown in the table.

**Table 9:** COMPASS WBS 6 costs in FY$22M

COMPASS Mission Estimate (FY22$M)			
WBS	Description	Total	Notes
6	Spacecraft	$263.3	Estimated via parametric models
6.1	Mechanical	$32.6	All subsystem estimates use the average of SEER Space and PRICE TruePlanning model outputs with the exception of three. The propulsion estimate is a ROM from the subsystem lead. FSW and Component Engineering estimates are based on average of larger APL historical missions.
6.2	Propulsion	$17.3	
6.3	Avionics	$35.2	
6.4	Power	$78.0	
6.5	Guidance & Control	$20.9	
6.6	Thermal	$4.9	
6.7	Telecommunications	$34.5	
6.8	Harness	$5.3	
6.9	Flight Software	$17.0	
6.A	Component Engineering	$17.7	

#### WBS 7 & 9 Mission Operations and Ground Data Systems

The COMPASS mission operations estimate includes mission operations planning and development, network security, data processing, and mission management. The pre- and post-launch mission operations estimate are based on the cost per month of NH, Dragonfly, Juno and OSIRIS-Rex. These missions represent expenditure on pre- and post-launch mission operations for projects of comparable scope and complexity. The ground data systesms estimate is a bottoms up estimate from a ground data systems subject matter expert. The COMPASS Ground Data system provides full life cycle support for Subsystem Test, Observatory I&T, Hardware Simulator Control, & Flight Operations. The cost estimate is based on extensive reuse of PSP, IMAP, and DART Ground Software via APL’s Mission Independent Ground Software (MIGS) as well as use of the existing Mission Operations Center (MOC).

#### WBS 8 Launch Vehicle & Service

The mission requires a launch vehicle that does not correspond with any of the options currently described in the Decadal Survey Ground Rules. As such, the figures used in this estimate are based on an evaluation of current best estimates of the cost of the capability that will be required. The price of a LV with Falcon Heavy Expendable-type capabilities, based on past pricing to NASA missions of EELVs, would be approximately $210M for a launch using a standard sized fairing.

#### WBS 10 System Integration and Testing

This element covers the efforts to assemble and test the spacecraft and instruments. The COMPASS I&T effort is estimated as 12.7% of the hardware. This percentage is based on a detailed analysis of cost actuals from previous APL missions, including MESSENGER, NH, STEREO, Van Allen Probes, and PSP. This percentage is allowed to vary along with hardware costs as part of the mission cost risk analysis to capture the risk historically manifested during I&T.

#### Deep Space Netowrk (DSN) Charges

This element provides for access to the DSN infrastructure needed to transmit and receive mission and scientific data. Mission charges are estimated at $28.5M using the Jet Propulsion Laboratory (JPL) DSN Aperture Fee tool. The DSN cost estimate covers pre- and post-contact activity for each linkage. The DSN aperture fee estimate is excluded from the mission budget and the cost tables in this report. DSN set up costs are estimated based on prior missions and included in the WBS 7 estimate.

### Confidence and Cost Reserves

**Fig. 19:**
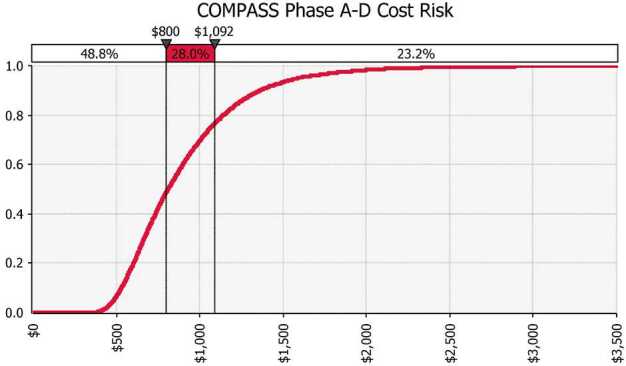
S-curve summary for COMPASS

The cost risk ranges by major WBS element as inputs for the COMPASS probabilistic cost risk analysis to quantify total cost risk are found in Tables 9 and 10 and are described below. The estimate includes unencumbered cost reserves of 50% of the estimated costs of all Phase A–D elements except for the launch vehicle. A probabilistic cost risk analysis shows 76.8% confidence that the Phase A–D mission is achievable within the estimated costs of this study (Fig. 19). The high confidence level is driven primarily by the large cost reserves for this pre-proposal concept. Given a typical competitive pre-Phase A NASA environment with 25% reserves on Phase A–D elements, the probabilistic cost risk analysis shows 64.7% confidence that the Phase A–D mission would be achievable. A 50th- to 70th-percentile confidence level is expected and reasonable for a pre-Phase A concept with this level of reserves.

**Table 10:** Cost risk analysis

Description	Value (FY$22M)	Confidence Level
Point Estimate	$799.9	48.8%
Mean	$899.3	
Standard Deviation	$374.7	
Reserves	$292.0	
Total w/ reserves	**$1091.9**	**76.8%**

A coefficient of variation (standard deviation/mean) of approximately 42% indicates appropriate levels of conservatism given the early formulation phase. The model confirms the point estimate and provides a reasonable basis for the COMPASS CML 4 study.

## Summary and Conclusions

COMPASS explores the fundamental mysteries that make Jupiter the greatest particle accelerator in our Solar System by visiting the heart of its radiation belts, which are regions largely avoided by other spacecraft due to the hazards they pose. Understanding how particles are accelerated to such high energies allow us to investigate a parameter space that is unattainable at Earth or elsewhere in the Solar System with the aim to bridge the knowledge gaps between Earth’s, planetary, and perhaps exosolar magnetospheres. Therefore, COMPASS aims to explore the distinctive and universal acceleration, source, transport, and loss processes for further our understanding of comparative processes.

The COMPASS mission concept presented here at CML 4, demonstrates that COMPASS is technically feasible, fully addresses its science objectives, and minimizes risk and cost of implementation. In summary, COMPASS is a single, solar powered spinner outfitted with comprehensive charged particle instrumentation that spans an unprecedented species and energy range, a magnetometer and plasma waves instrument suite to diagnose the full wave spectrum with multidirectional antennas, and the first-ever dedicated X-ray imager. COMPASS can be delivered to the Jupiter system via an expendable Falcon Heavy launch vehicle with $\Delta $V-Earth gravity assist (EGA) trajectory and an interplanetary cruise time of flight of 5.5 – 6 years. The prime science mission consists of a multiple phased approach to mitigate the effects of Jupiter’s intense radiation environment, while still enabling critical observations into the most-intense radiation belts in the Solar System. Altogether, the prime mission comprises 15 orbits spanning ∼1.5 years. The full life cycle cost (Phases A-F; with 50% unencumbered reserves, including the launch vehicle) is ∼$1.2B (FY22$).

## Figures and Tables

**Fig. 20:**
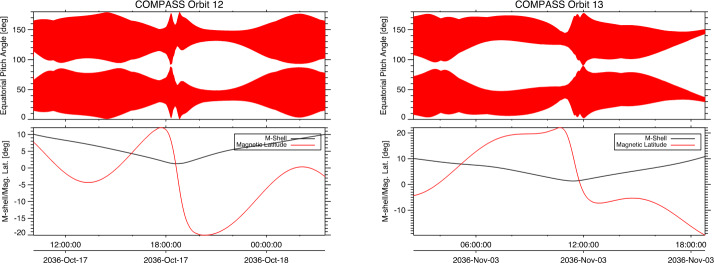
Upper panels: Equatorial pitch angle coverage by a single telescope instrument mounted perpendicular to the spacecraft spin plane for orbits 12 & 13. Lower panels: Location of COMPASS in L-shell and magnetic latitude, calculated in the JRM09+CAN magnetic field (Connerney et al. 1981, 2018). The two panels show two example orbits in science phase 2 where pitch angle coverage is most critical. It can be seen that coverage is not continuous, but full equatorial coverage can be accomplished by combining neighboring orbits

**Fig. 21:**
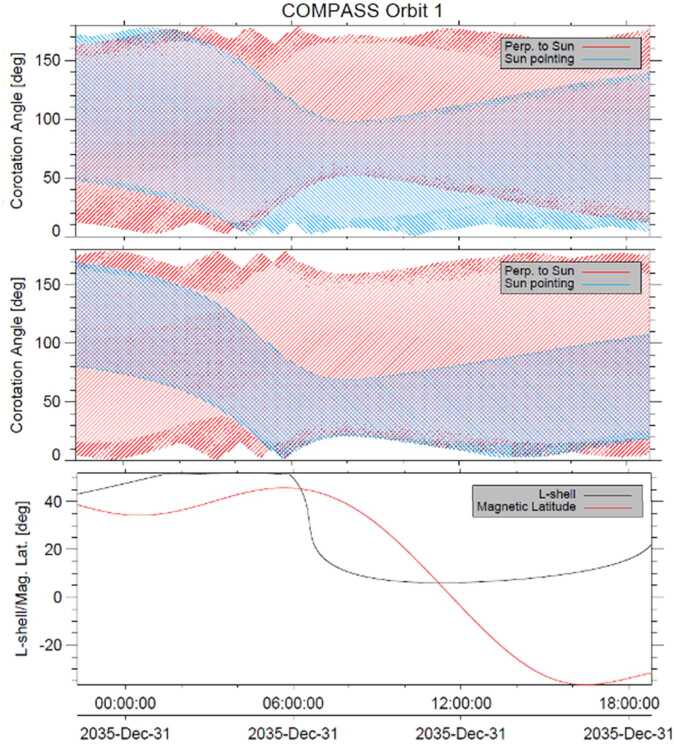
Coverage of the corotation direction using two TPD instruments (red and blue) illustrated based on the example of orbit 1. Upper panel: baseline mission with two instruments that are tilted 30deg from the spin axis. It can be seen that they cover the corotation direction at all times. Further optimization may be possible to extend the coverage toward the <10deg directions. Middle panel: The two instruments are pointed perpendicular and parallel to the spin axis. It can be seen that the perpendicular instrument alone is able to cover almost directions and that the parallel one can be descoped if necessary. Lower panel: L-Shell and magnetic-latitude coverage of COMPASS in orbit 1

**Fig. 22:**
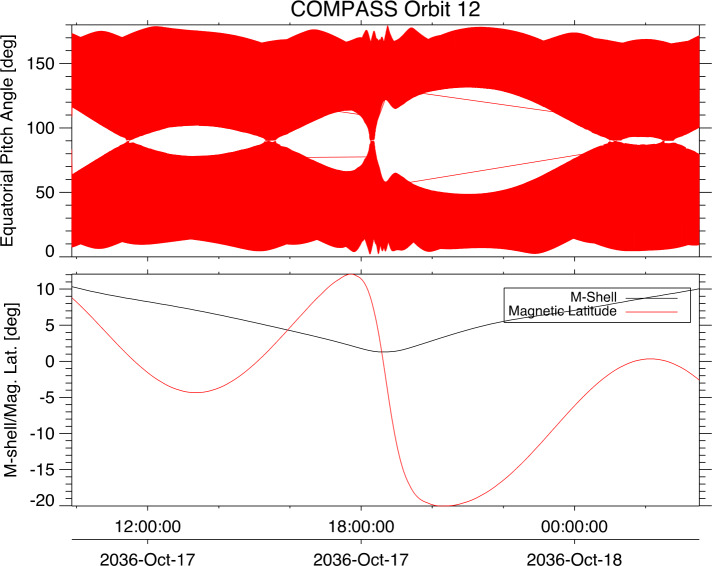
Similar format as Fig. 20, but instead using a multi-look direction instrument such as Juno/JEDI

**Fig. 23:**
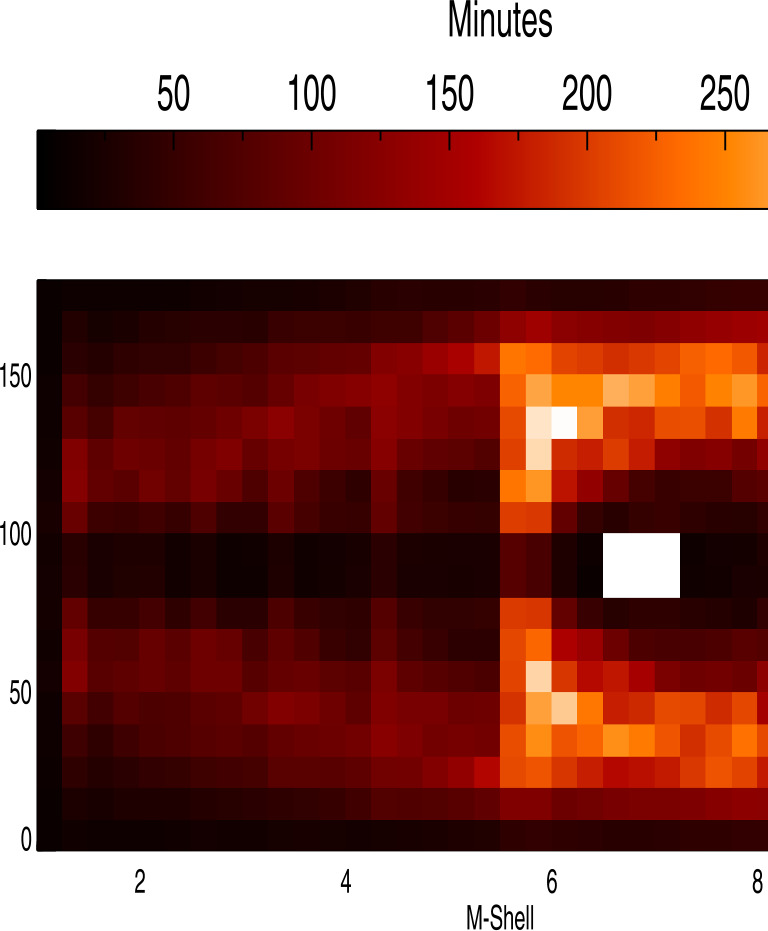
Color coded sampling durations as a function of equatorial pitch angle and M-shell for all COMPASS orbits

**Fig. 24:**
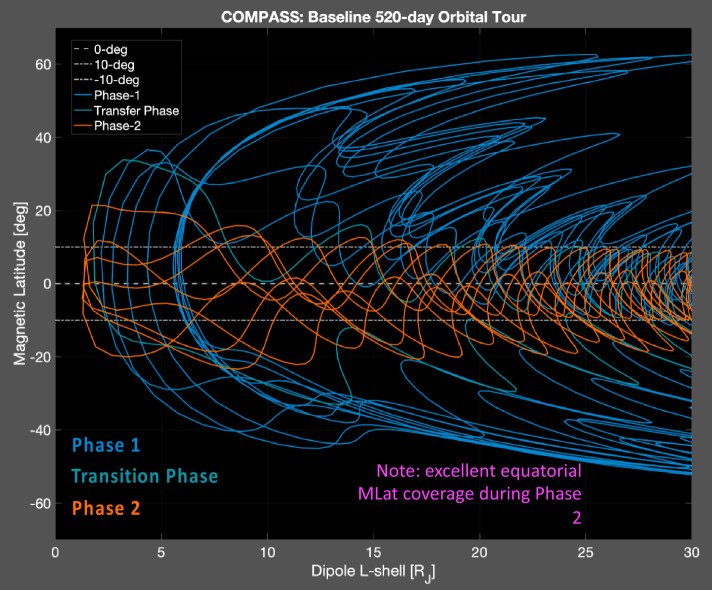
Magnetic latitude coverage of COMPASS for science phase 1 (blue trajectories) and science phase 2 (orange trajectories)

**Fig. 25:**
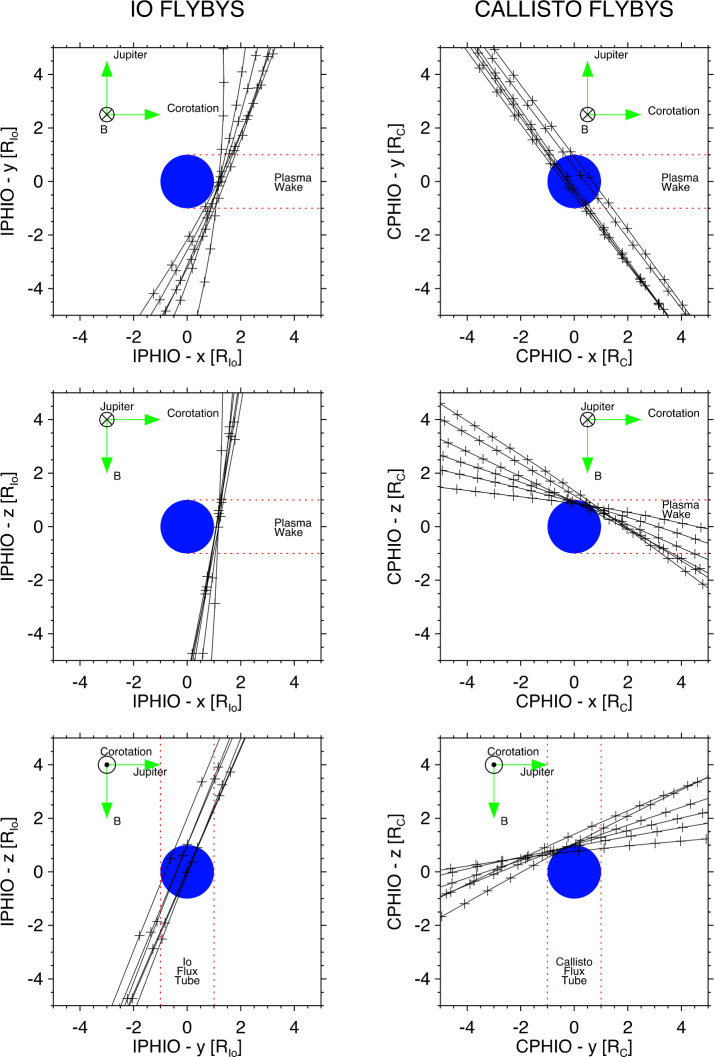
COMPASS flyby trajectories in a moon-centered coordinate system for both Io (left panels) and Callisto (right panels). Dashed red lines illustrate the approximate geometrical wake and flux tube locations. In reality, these are tilted and depend on the moon’s location with respect to the Jovian current sheet, but this is not taken into account here

**Fig. 26:**
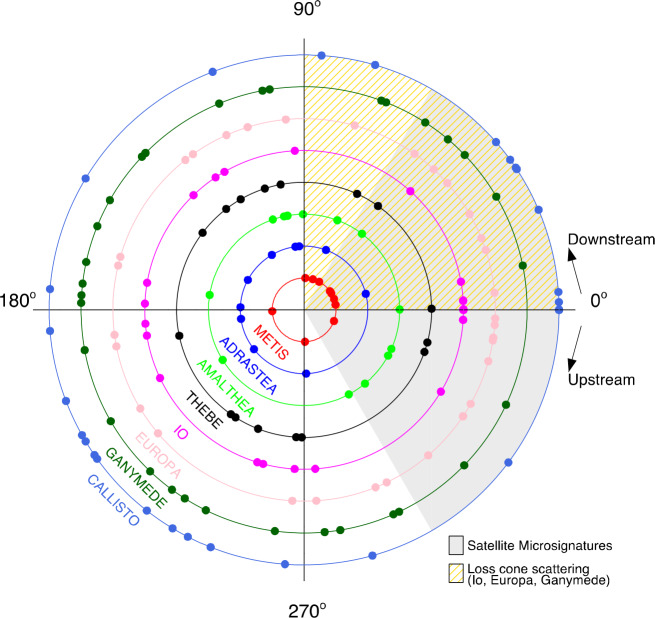
Polar projection illustrating COMPASS’ longitudinal coverage of the various Jovian moons. In addition to the Galilean moons, COMPASS will make repeated passes of the inner-most moons that are relatively unexplored. The shaded regions highlight a set of longitudes where we expect to carry out observations associated with satellite microsignatures (gray shading) and processes associated with loss cone scattering (beige shading). Dots represent the longitudinal positions of the moons during times when COMPASS crosses magnetic field lines that map to their orbital regions

## Appendix A: Pitch Angle and Corotation Coverage

Charged particles bounce along magnetic field lines depending on their equatorial pitch angle. Energetic ions and all electrons organize mostly with pitch angle. Low energy plasma on the other hand flows along the corotation direction. In order to fully characterize the particle distributions, COMPASS needs to be able to cover a large range of equatorial pitch angle and at least the nominal corotation direction. Equatorial pitch angle coverage at Jupiter is always a challenge because the magnetic and rotational equator planes are tilted, meaning that a spacecraft orbit never stays in the magnetic equator, and because the magnetic field lines are stretched tail-like already in the middle magnetosphere (Connerney et al. 2022), meaning that even small magnetic latitude limit the equatorial pitch angle coverage. Figure 20 shows that our chosen tour still manages to build up full equatorial pitch angle coverage—assuming a single look direction (or telescope) instrument—through combining neighboring orbits. Instantaneous pitch angle gaps will be mitigated by filling them with information from other orbits (as in Smirnov et al. 2022). Coverage of the nominal corotation direction would be patchy when using a single instrument. Different to the equatorial pitch angle coverage that mostly depends on the magnetic latitude, the local coverage of the corotation direction can be fixed through adding another instrument (Fig. 21), which is why we baseline two plasma sensors.

Instruments with multiple instantaneous look directions (such as Juno/JEDI, Mauk et al. 2017) can obtain more complete instantaneous pitch angle coverage (Fig. 22). And finally, Fig. 23 illustrates the sampling duration of equatorial pitch angles as a function of M-shell (using the Connerney et al. 2018 magnetic field model) for all COMPASS orbits.

## Appendix B: Magnetic Latitude Coverage

An important requirement for COMPASS is to ensure complete PAD coverage (see discussion above) to reveal acceleration, loss, and transport processes in the inner-most regions. Therefore, Jovigraphic and magnetic latitude requirements were imposed on the design tour. Figure 24 illustrates the magnetic latitude coverage of COMPASS over both science phases as well as the transition phase. In the second science phase, it can be seen that COMPASS is able to skirt along magnetic latitudes < 10^∘^, which is critically important.

## Appendix C: Spatial Coverage Near the Jovian Moons

Repeated Io and Callisto flybys are utilized to reduce the orbit period and apojove as well as reduce the inclination and perijove distances. Although the flybys are not tied to the baseline or threshold science requirements, there does exist many enhancing, cross-divisional, science opportunities. For example, the X-ray imager on board COMPASS is capable of measuring fluorescence emitted from the moon’s surfaces. Figures 25 and 26 illustrate the flyby trajectories in a moon-centered coordinate system as well as the longitudinal coverage, respectively.
